# Synthesis and Biological Aspects of Mycolic Acids: An Important Target Against *Mycobacterium tuberculosis*

**DOI:** 10.1100/tsw.2008.99

**Published:** 2008-07-31

**Authors:** Marcus Vinícius Nora de Souza, Marcelle de Lima Ferreira, Alessandra Campbell Pinheiro, Maurício Frota Saraiva, Mauro Vieira de Almeida, Marcelo Siqueira Valle

**Affiliations:** ^1^ Instituto de Tecnologia em Fármacos-Far-Manguinhos, Rua Sizenando Nabuco, 100, Manguinhos, 21041-250 Rio de Janeiro, RJ, Brazil; ^2^ Instituto de Química Universidade Federal Fluminense, Campus do Valonguinho S/n Centro 24020-150 Niterói, RJ, Brazil; ^3^ Instituto de Química, Universidade Federal do Rio de Janeiro, 21941-590 Rio de Janeiro, RJ, Brazil; ^4^ Departamento de Química, Universidade Federal de Juiz de Fora, 36036-330 Juiz de Fora, MG, Brazil

**Keywords:** tuberculosis, mycolic acid, drug discovery

## Abstract

Mycolic acids are an important class of compounds, basically found in the cell walls of a group of bacteria known as mycolata taxon, exemplified by the most famous bacteria of this group, the *Mycobacterium tuberculosis* (*M. tb.*), the agent responsible for the disease known as tuberculosis (TB). Mycolic acids are important for the survival of M. tb. For example, they are able to help fight against hydrophobic drugs and dehydration, and also allow this bacterium to be more effective in the host's immune system by growing inside macrophages. Due to the importance of the mycolic acids for maintenance of the integrity of the mycobacterial cell wall, these compounds become attractive cellular targets for the development of novel drugs against TB. In this context, the aim of this article is to highlight the importance of mycolic acids in drug discovery.

## INTRODUCTION

The mycobacterial cell wall is very important as a cellular component surrounding the cell membrane, providing additional support and protection. Basically, it is comprised of three covalently linked substructures: mycolic acids, peptidoglycan, and arabinogalactan, which represent over 60% of cell dry weight[1]. Mycolic acids are an important class of compounds, basically found in the cell walls of a group of bacteria known as mycolata taxon, exemplified by the most famous bacteria of this group, the *Mycobacterium tuberculosis* (*M. tb.*)**, the agent responsible for the disease known as tuberculosis (TB)[2]. Mycolic acids are important for the survival of *M. tb.* For example, they are able to help fight against hydrophobic drugs and dehydration, and also allow this bacterium to be more effective in the host's immune system by growing inside macrophages[3]. Structurally, mycolic acids are comprised of long fatty acids containing different functional groups, such as double bonds, keto, ester, epoxy, methoxy, and cyclopropane ring. They possess a broad family of over 500 species, which are commonly characterized by the numbers of carbon atoms presented in the fatty acids[1,4]. For example, mycolic acids, which possess 60–90 carbon atoms, are commonly isolated from *Mycobacterium* and they are called mycolic or eumycolic acids. In the case of the mycolic acids containing carbon atoms between 22–60 units, they are isolated from other species, such as *Nocardia* and *Corynobacterium*, and they are named nocardomycolic and corynomycolic acids, which contain 44–60 and 22–36 carbon atoms, respectively[1,4]. Fig. 1 shows some compounds of the mycolic acid class; for example, compound **1**, which is a key structural component of the cell envelope of *M. tb.*, and other compounds present in different mycobacterial cell walls, such as α-methyl-trans-cyclopropanes (α-mycolic acids) **2**, α-methyl-β-methoxy groups (methoxymycolates) **3**, α-methyl-β-keto groups (ketomycolates) **4**, and α-methyl-trans-epoxy groups **5**.

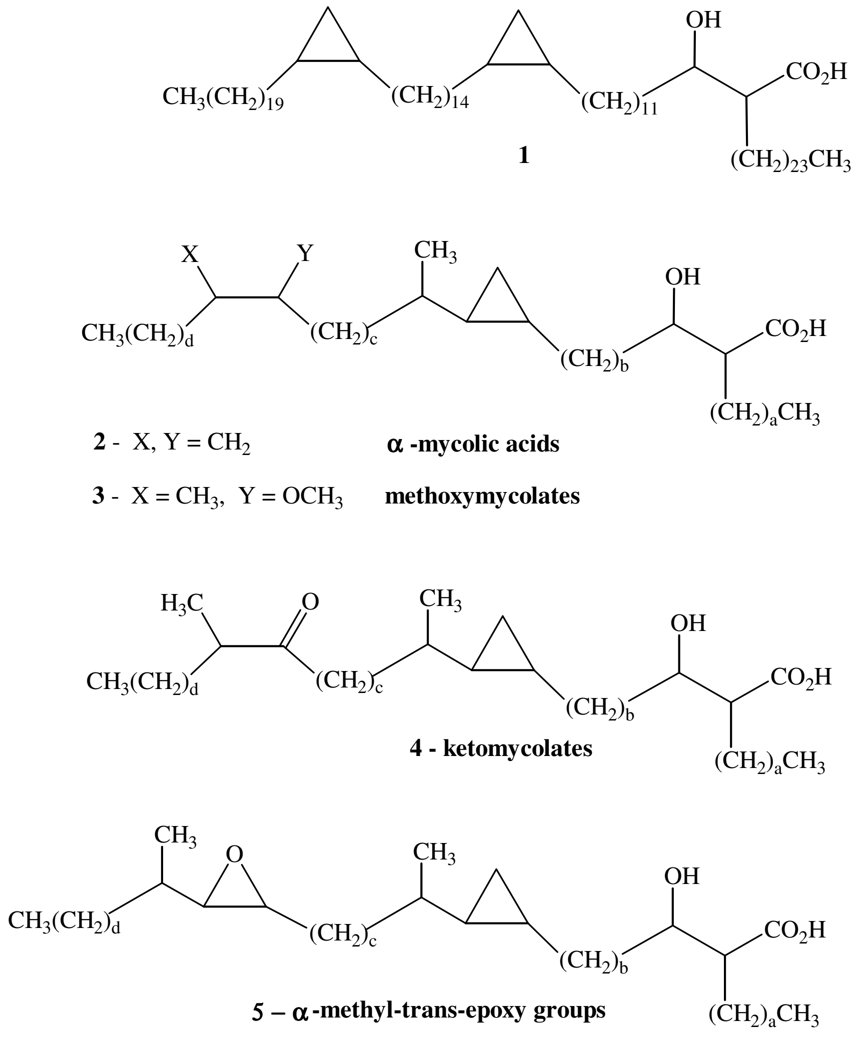

**FIGURE 1.** Some compounds of the mycolic acid class.

Mycolic acids were isolated for the first time in 1938 by Andersen and coworkers at the Chemistry Department of Yale University, from an extract of human *M. tb.*, which after heating under reduced pressure and high temperatures, furnished the oil hexacosanoic acid in 24% yield[5]. However, despite the importance of this work, the mycolic acid structure was only elucidated 12 years later by Asselineau, which demonstrated that mycolic acids were comprised of a β-hydroxy-α-alkyl branched chain[6]. The structure of α-mycolic acids is divided into two parts: the mycolic motif, comprised of the β-hydroxy-α-alkyl branched chain, and the long alkyl chain named meromycolate moiety **6** (for alkyl groups, which possess 50 or more carbon atoms). The cleavage by thermolysis applied by Andersen and coworkers in 1938[5] of hydroxyacid functionality, producing the meromycolaldehyde **7** (Scheme 1), is still a standard procedure for the characterization of mycolic acids.

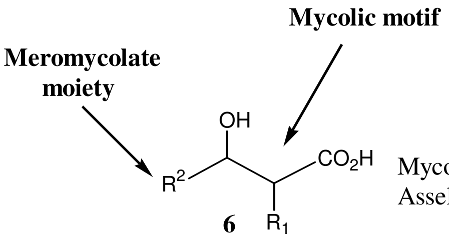

Mycolic acid structure proposed by Asselineau in 1950.

$R^1$ = long alkyl chain with different functional groups
$R^2$ = may or may not contain another oxygenated group, such as hydroxyl, methoxyl, keto, or carboxyl group.

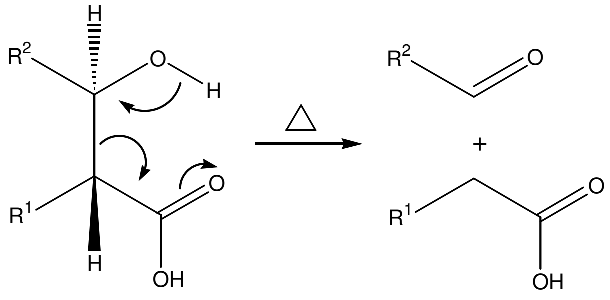

**7**

**8**

**6**

**SCHEME 1.** The structure of α-mycolic acids and the cleavage by thermolysis of hydroxyacid functionality, producing the meromycolaldehyde **7.**

During the period of 1950–1960, with the development of chromatography techniques, a variety of mycolic acid structures were identified in different mycobacteria strains. However, a more precise structural definition of the different species of mycolic acids started during the period of 1960–1970, with the use of NMR and MS analyses. During the period of 1970–1980, little scientific progress was made in the mycolic acid field, perhaps due to the fact that TB was almost eradicated. However, after 1980, TB again became a worldwide problem due to the AIDS epidemic, the advent of multidrug resistant (MDR) strains, and the lack of new drugs in the market[7]. In this context, the mycobacterial cell wall regained attention, and today there is a better comprehension of different aspects of the cell wall of *M. tb.*, such as structure, biosynthesis, and genetics[2], due to modern analysis techniques and the elucidation of the *M. tb.* Genome.

Due to the importance of the mycolic acids for maintenance of the integrity of the mycobacterial cell wall, these compounds become attractive cellular targets for the development of novel drugs against TB because the drugs would be capable of acting on proteins or enzymes encoded for specific genes of *M. tb.* In this context, the aim of this article is to highlight the importance of mycolic acids in drug discovery.

## BIOSYNTHESIS OF MYCOLIC ACIDS

The knowledge concerning the biosynthesis of mycolic acids is very important, both for the discovery of new therapeutic targets against TB and for the briefing of the mechanisms of action of the drugs currently used[8]. In this context, in general, biosynthesis of fatty acids is produced through some cycles of reactions, as condensations, dehydratations, keto reductions, and enoyl reductions. In this process, an enzyme series, such as β-ketoacyl synthase (KAS), β-ketoacyl reductase (KR), β-hydroxyacyl dehydrase (DE), and enoyl reductase (ER), is involved. More specifically, there are two types of fatty acid synthase systems (FAS): the multifunctional polypeptide FAS I system, which promotes the synthesis *de novo* of fatty acids, generating precursors of 14–26 carbon atoms; and the FAS II system, which includes monofunctional enzymes, which elongate the products of FAS I to give rise to precursors of mycolic acids of long chain[2,9].

Scheme 2 shows the main steps of the FAS I system, in which it can be evidenced that an acetyl group is elongated by two carbon units, using acetyl-CoA and malonyl-CoA as substrates to yield butyryl-S-Enz. This cycle is repeated, generating derivatives with 16 and 18 carbon atoms, used for the synthesis of membrane phospholipids; others with 20 carbons (start point where the FAS II systems take over for the synthesis of the very-long-chain mero segment of α-, methoxy-, and keto-mycolic acids) and 26 carbons, which becomes the short α-alkyl chain and methyl carboxyl segment of all mycolic acids of *M. tb.*[10].

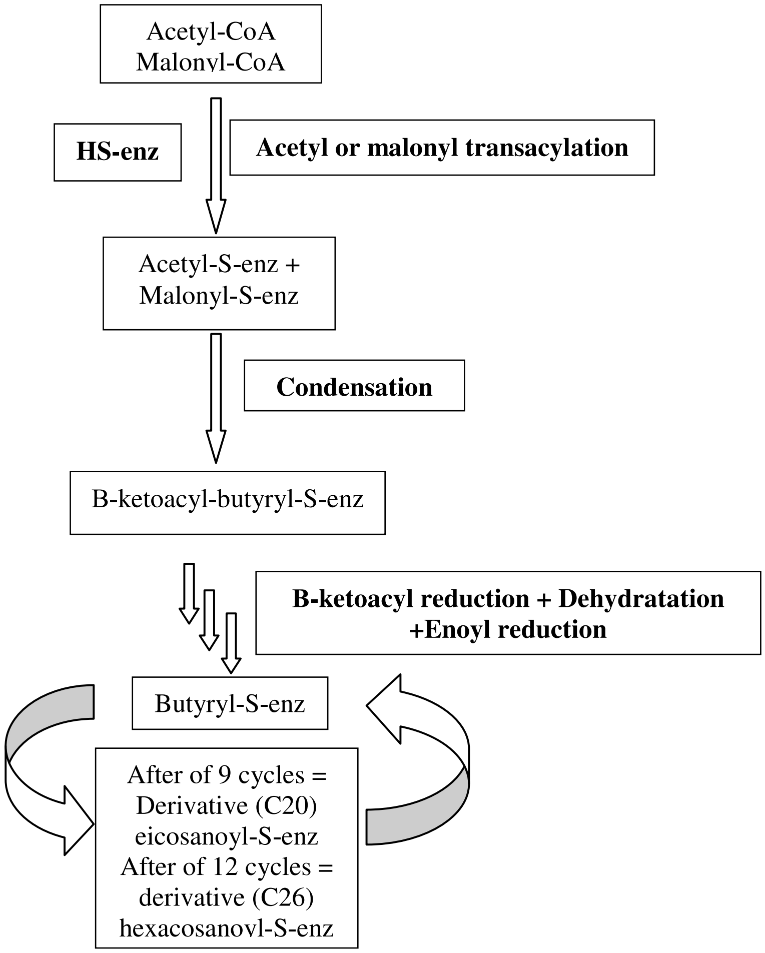

**SCHEME 2.** Steps of FAS I system that generates the derivatives eicosanoyl-S-enz and hexacosanoyl-S-enz. These fatty acids are released as the CoA derivatives and they will be used as precursors in FAS II.

After the synthesis of precursors, FAS II is started (Scheme 3). This system is formed of dissociable enzyme components, which act on a substrate bound to an ACP (acyl carrier protein). The first step consists of a reaction that aims to convert the produced precursors **11** and **12** in FAS I to β-ketoacyl derivatives **13** through an enzyme called β-ketoacyl-ACP-synthase (encoded by *KasA/KasB* gene)[11]. This reaction uses as cosubstrate a molecule of malonyl-S-ACP **10**, which is derived from the reaction between malonyl-S-CoA **9** and the enzyme malonyl-CoA ACP transacylase, which is encoded by *FabD* gene[12,13]. The second step involves the reduction of the product **13** obtained in the last step by enzyme β-ketoacyl-ACP-reductase, which furnished the intermediate **14**. This product is then converted to unsaturated derivative **15** by β-hydroxyacyl-ACP-dehydratase. Compound **15** is reduced by 2-*trans*-enoyl-ACP reductase (encoded by *InhA* gene) to yield the final product **16**, which is two carbons longer than the starting substrate **11** and **12**, which can be further recycled to produce others derivatives with chains increased by two carbon atoms[14,15].

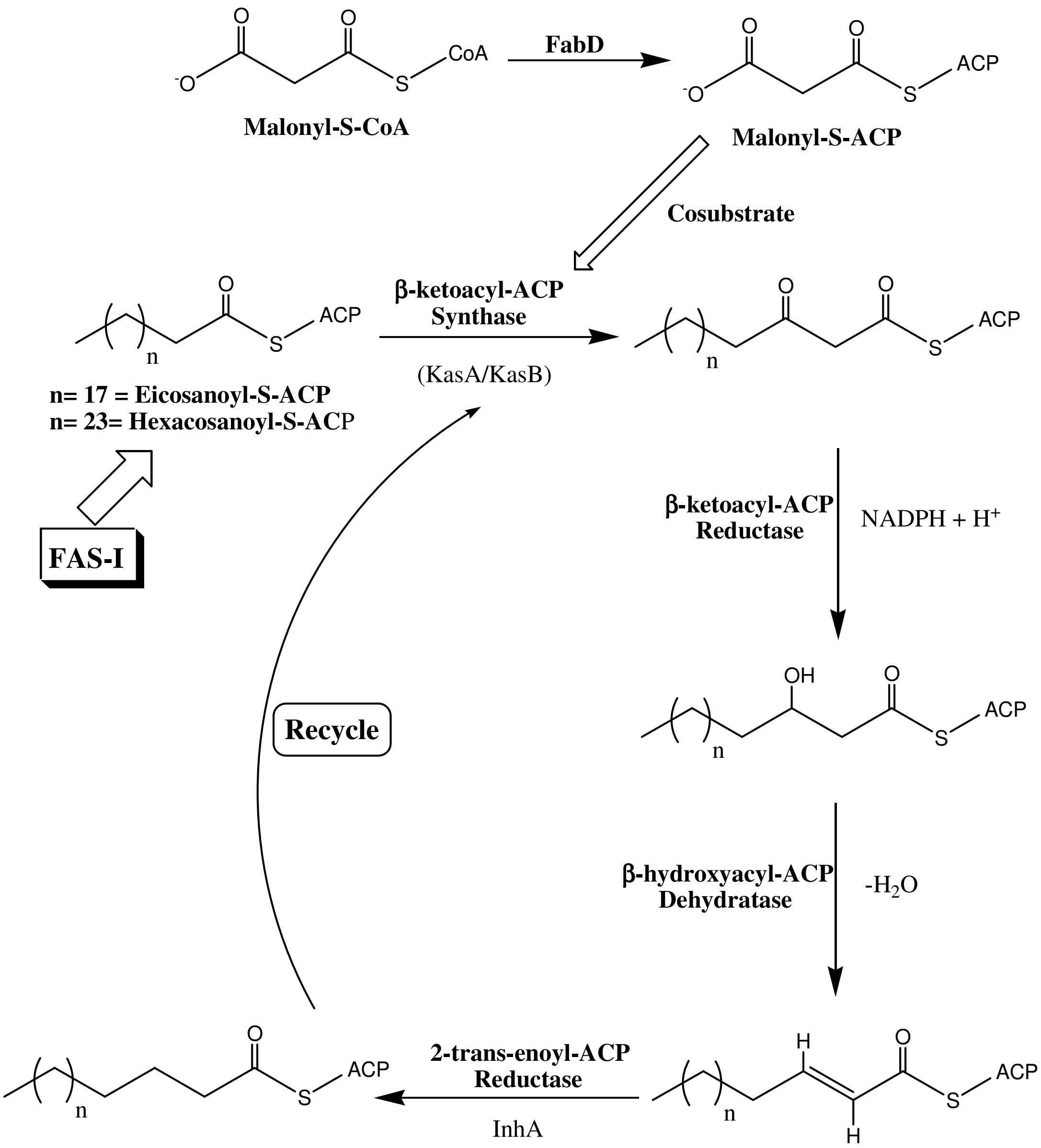

**SCHEME 3.** Elongation of fatty acids by FAS II, which produces long-chain mycolic acid derivatives.

Subsequently, the introduction of functional groups into the mero chain is realized through desaturation of saturated alkyl chain **17** to yield two *cis* double bonds **18**, which are converted easily to others groups as necessary. This reaction occurs through the action of an aerobic terminal, mixed function enzyme called desaturase, which promotes the oxidation of saturated **17** to yield unsaturated fatty acid **18** with the coincident reduction of molecular oxygen and oxidation of NADPH.

Others derivatives can be synthesized from the unsaturated fatty acid **18** formed in the desaturation reaction, e.g., containing methoxy and keto moieties, the methyl branches adjacent to *trans*-olefins and the bridging methylenes of the cyclopropane rings. These derivatives are all derived from methionine, via S-adenosyl-L-methionine (SAM) **19**, which adds a methyl group to the unsaturated derivative, originating a carbonium ion **20,** which is the key intermediate for the others transformations[2,16] (Scheme 4).

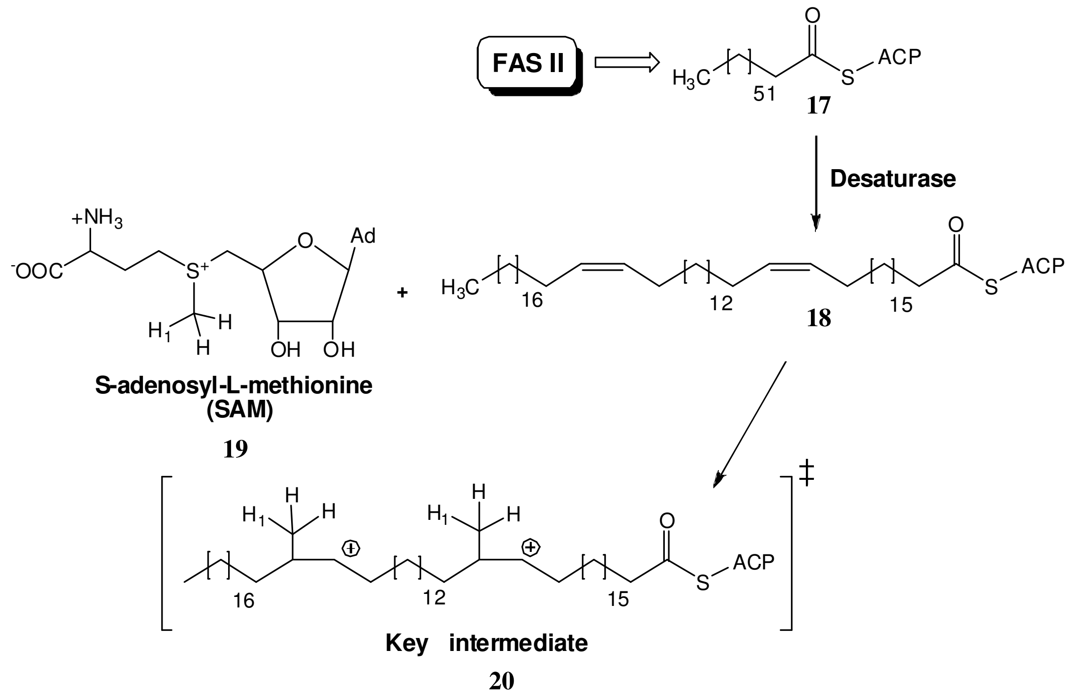

**SCHEME 4.** Formation of key intermediate (carbonium ion) that will be converted into other functionalized derivatives.

After the formation of the intermediate **20**, other SAM-dependent methyl transferase enzymes act to yield other desired derivatives (Table 1 and Scheme 5). Table 1 shows the genes and the corresponding proteins responsible for the preparation of derivatives **21–25**[17,18,19,20,21].

After these modifications, the β-hydroxy acid moiety is introduced through a biological equivalent mechanism to a Claisen-Condensation (Scheme 6). This process starts with the conversion of the derivatives **12** and **22** generated from the FAS I and FAS II processes, respectively. Thus, the hexacosanoyl derivative ($C_{26}$-S-CoA) **12** is converted to the 2-carboxyl-$C_{26}$-CoA **26** by acyl-CoA carboxylases ($AccD_4$ and $AccD_5$). The α-meroacyl-S-ACP **22** derivative is transformed to α-meroacyl-AMP **27** by the enzyme called FadD32. These two formed products are the substrates for the condensation reaction catalyzed by the complex enzyme Pks13 (polyketide synthase 13). This enzyme has five domains: PPB (two nonequivalent domains), KS, AT, and TE. First, the α-meroacyl-S-ACP **22** and 2-carboxyl-$C_{26}$-CoA **26** are covalently attached to each one of the PPB domains, which are separate for the AT and KS domains. After that, the meroacyl group is transferred to the KS domain, where the condensation reaction occurs. This step is followed by the reduction of the 3-oxo group to the secondary alcohol by an unknown reductase to yield the mature α-mycolate[22].

**TABLE 1**
**Responsible Genes and Proteins for Modifications in the Mero Chain, after the Desaturation Reaction**

| Genes | Proteins | Functions |
|---|---|---|
| *cma1* | CMAS-1 | Introduces a cyclopropane ring at the distal position. |
| *cma2* | CMAS-2 | Introduces a cyclopropane ring at the proximal position. It has specificity for *cis* double bonds. |
| *mma4* | MMAS-4 | Introduces the hydroxyl group with an adjacent methyl branch at the distal position. |
| *mma3* | MMAS-3 | Converts the hydroxyl group to form a methyl ether. |
| *mma2* | MMAS-2 | It is similar to CMAS-2, but MMAS-2 introduces a cyclopropane ring in oxygenated series of mycolates while CMAS-2 acts in the α-series. |
| *mma1* | MMAS-1 | Converts the proximal *cis* double to a *trans* double bonds with an allylic methyl branch. |

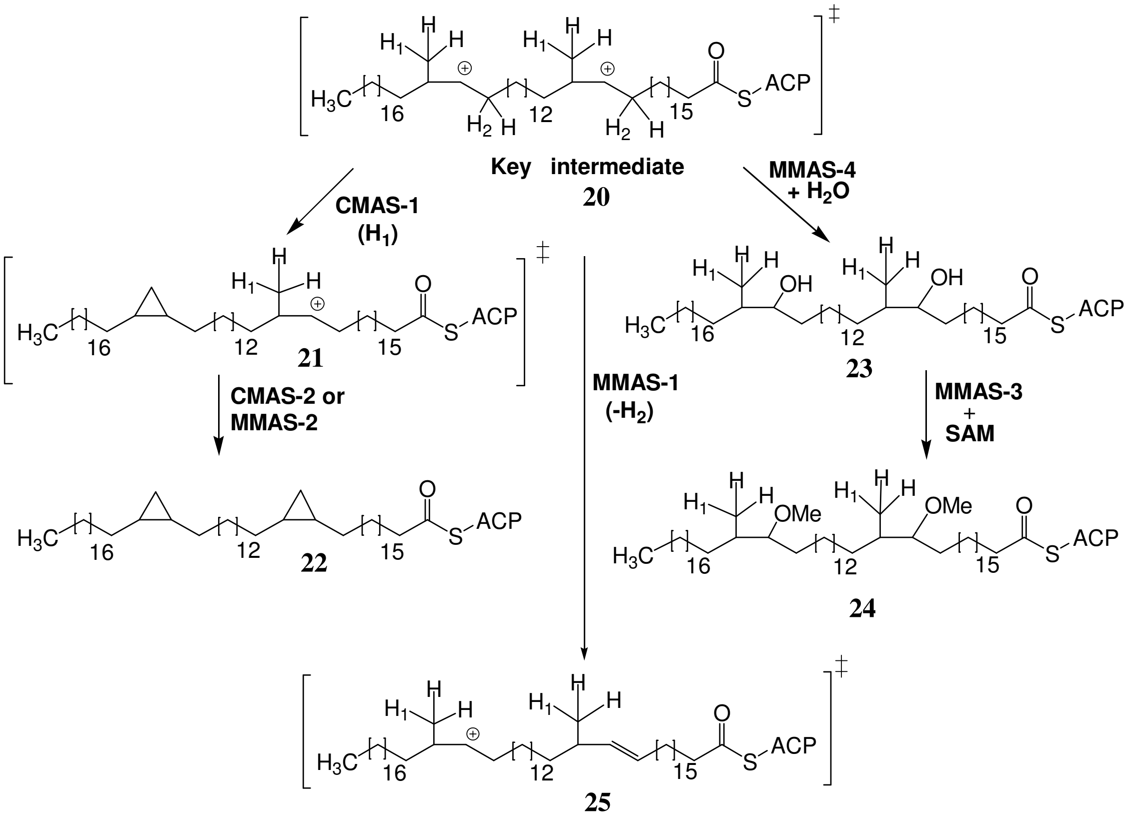

**SCHEME 5.** Preparation of important derivatives **21–25** by SAM-dependent methyl transferase enzymes.

At the end of the synthesis, transport of newly synthesized mycolic acids followed by attachment to the peptidoglycan-arabinogalactan complex and the formation of TDM (trehalose dimycolate) **33** are produced. This step is not well known, only the mycolyltransferase function of antigen 85 (Ag85) is well established. Therefore, many researchers have been studying this pathway. Takayama and coworkers suggested a completely new pathway to the synthesis of TMM **32**, which is described in Scheme 7[10,23,24,25]. In this scheme, the final product of the synthesis of the mycolic acids is found to be attached to Pks 13. The mycolyl group is transferred to D-mannopyranosyl-1-phosphoheptaprenol **28** to

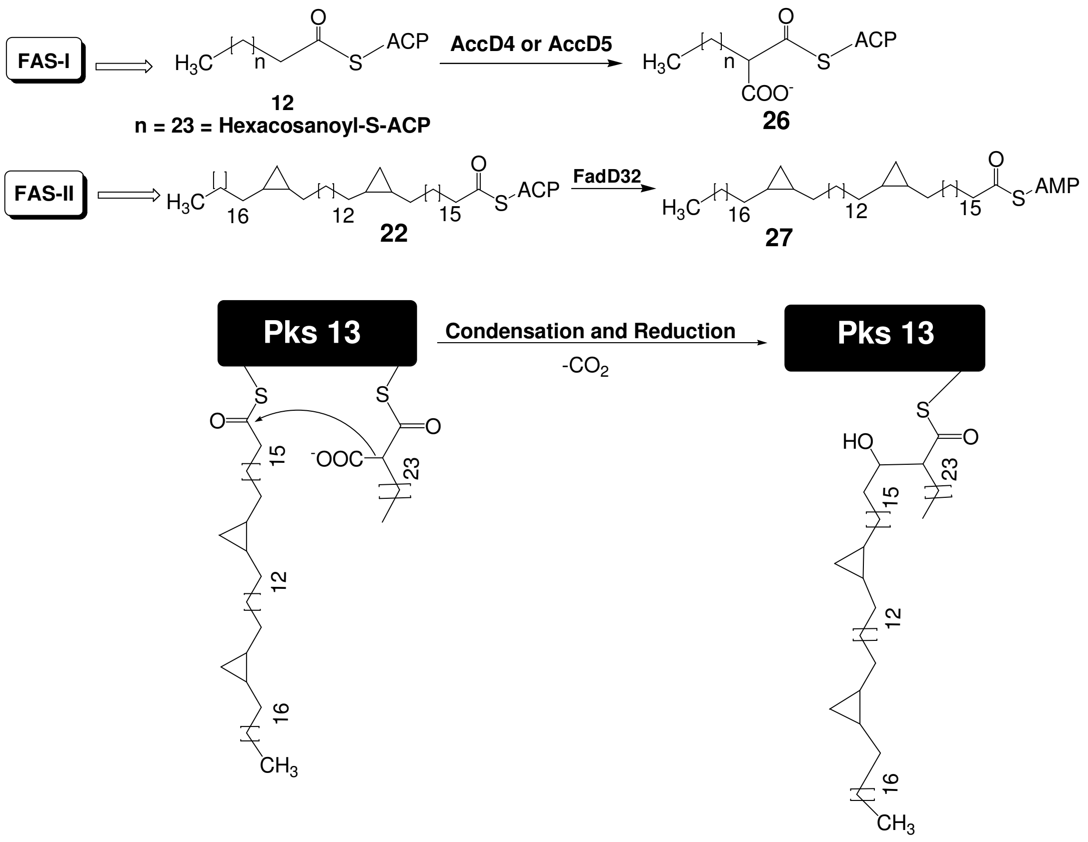

**SCHEME 6.** Synthesis of α-mycolic acid by Pks 13 is shown in a simplified way. First, the products **12** and **22** derived from FAS I and FAS II, respectively, are converted to 2-carboxyl-$C_{26}$-CoA **26** and α-meroacyl-AMP **27**, respectively. Further, these derivatives are covalently attached to Pks 13, which is processed via the condensation reaction, followed by a reduction of the 3-oxo group to the secondary alcohol.

yield Myc-PL (6-O-mycolyl-β-D-mannopyranosyl-1-phosphoheptaprenol) **29**. This reaction is catalyzed by a proposed cytoplasmatic mycolyl transferase I, followed by the Myc-PL, which migrates to the surface of the cell membrane and interacts with the ABC transporter, where the three last reactions are processed. In the first step, the mycolyl group is transferred to trehalose-6-phosphate **30** by mycolyl transferase II to yield 6-O-mycolyl-treh-6'-P **31**. In the second step, the dephosphorylation is promoted by TMM-P-phosphatase to form TMM **32**. Finally, in the last step, substance **32** is transferred outside the cell through the ABC transporter, where it is involved in the synthesis of both TDM **33** and cell wall arabinogalactan-mycolate (AG-M). These processes are mediated by Ag 85 complex.

## PEPTIDOGLYCAN AND ARABINOGALACTAN

Peptidoglycan and arabinogalactan are also important polysaccharides in the mycobacterial cell walls, which are linked by covalent attachment to arabinogalactan (AG), a polymer composed primarily of D-arabinofuranosyl and D-galactofuranosyl residues, attached to mycolic acids and peptidoglycan and is known as mycolyl-arabinogalactan-peptidoglycan (mAGP) complex (Scheme 8)[26]. The biosynthesis of this complex involves different enzymes, such as galactopyranosyl mutase and epimerases, and arabinosyl and galactofuranosyl transferases.

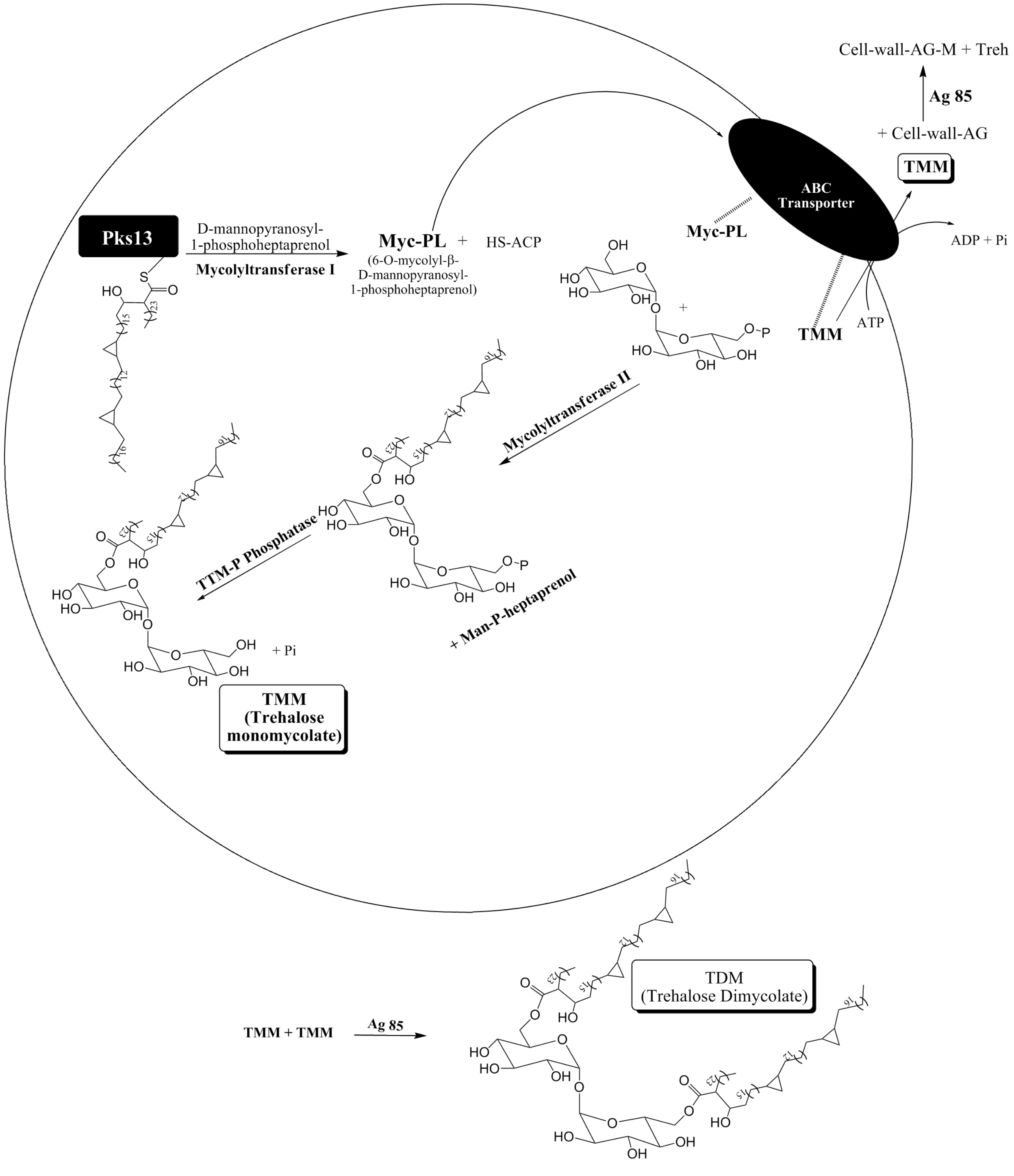

**SCHEME 7.** Transport of newly synthesized mycolic acids followed by attachment to the peptidoglycan-arabinogalactan complex and the formation of TDM **33.**

The mycobacterial cell wall is also comprised of 6,6´-dimycolyltrehalose **34** (Fig. 2), biosynthesized by three homologous proteins, Ag85, A, B, and C, which possess mycolyltransferase. The antigen 85 (Ag 85) complex is a major protein component of the mycobacterial cell wall, all of which contribute to cell wall biosynthesis and help to maintain its integrity, catalyzing the transfer of mycolic acids into the envelope[27].

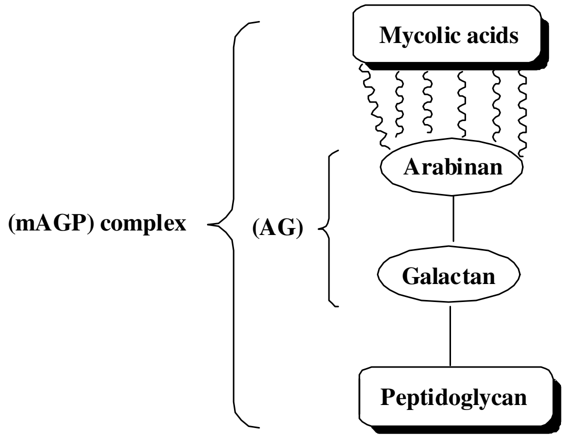

**SCHEME 8.** Mycolyl-arabinogalactan-peptidoglycan (mAGP) complex.

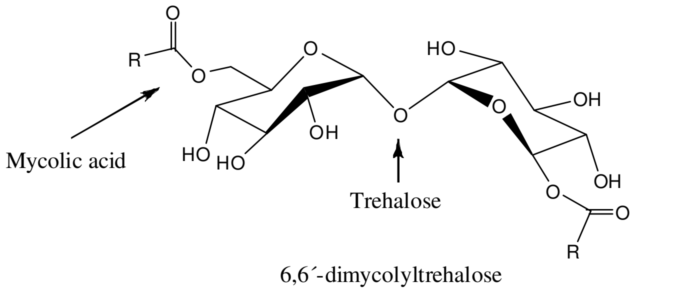

**34**

**FIGURE 2.** Structure of 6,6´-dimycolyltrehalose **34.**

## DRUGS THAT INHIBIT THE BIOSYNTHESIS OF *MYCOBACTERIUM TUBERCULOSIS´S* CELL WALL

Nowadays, there are two prodrugs that inhibit specific enzymes involved in the biosynthesis of these constituents of the cell wall: isoniazid and ethionamide (Fig. 3).

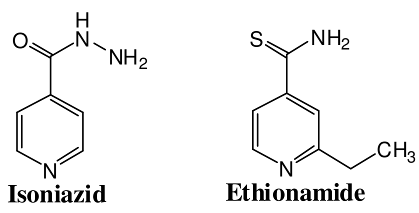

**FIGURE 3.** Structures of isoniazid and ethionamide.

## Isoniazid (INH)

INH was synthesized, for the first time, in 1912, for the Czech researchers Meyer and Malley as part of their doctoral work in Prague. However, only 4 decades later, after investigations at pharmaceutical companies Hoffman La Roche, Farbenfabriken Bayer, and Squibb Institute for Medical Research, each independently discovered INH as an anti-TB agent. The first clinical trial began in 1951 at Sea View Hospital in Staten Island, NY and was reported to the public in 1952[28].

INH is one of the most powerful synthetic agents against the *M. tb.* complex and its minimum inhibitory concentration (MIC) is very low (0.1–0.5 µg/ml). This high potency can be justified for the drug to possess many targets inside of the cell. However, the mode of action of this compound is still not completely understood.

The probable mechanism that is more accepted involves the inhibition of biosynthesis of mycolic acids, which compose the mycobacterial cell wall (Scheme 9)[29]. However, before that can happen, it is known that INH needs a previous *in vivo* activation to exercise its anti-TB activity. The responsible enzyme for this function is called KatG, which has dual activities of catalase and peroxidase. This enzyme has two identical subunits of 80 kDa and protects the *M. tb.* of the noxious lesion caused by hydrogen peroxide generated during the oxidative metabolism[30,31].

The activation of INH through this enzyme can generate isonicotinic acid, acyl radicals, peracids, and aldehydes capable of reacting with nucleophilic groups present inside the cell, e.g., the formation of the isonicotinoyl radical **37,** which reacts with the nicotinamide group of NAD (nicotinamide adenine dinucleotide) to yield the INH-NAD adduct **38**[32,33]. This adduct inhibits and binds to enzymes involved in the biosynthesis of mycolic acids, resulting in cell death. The main enzyme inhibited by the binding of the INH-NAD adduct is called *trans*-2-enoyl-ACP reductase, encoded by the *InhA* gene. This enzyme is an enoyl reductase that catalyzes the NADH-dependent reduction of long-chain *trans*-2-enoyl-ACP, in the last step of FAS II, promoting the stop of the elongation phase of mycolic acids and cell lysis[34].

## Ethionamide (ETH)

ETH, a structural analogue of INH, is only used in case of bankruptcy of the front-line drugs. It is the most frequently used drug for the treatment of drug-resistant TB. ETH, like INH, is a prodrug, which needs a previous activation to inhibit the synthesis of mycolic acids, leading to bactericidal activity[35,36,37] (Scheme 9). The responsible enzyme for this activation is a monooxygenase called EthA. Then, the activated ETH **40** reacts with $NAD^+$ to yield an ETH-NAD **42** adduct that had been hypothesized to be the inhibitor of *InhA*[38] (Scheme 9).

## Ethambutol (ETM)

Besides mycolic acids, others constituents of the mycobacterial cell wall are presented as interesting cell targets in the combat to the Koch's bacillus, e.g., the arabinogalactan (AG), an important polysaccharide that forms the mAGP complex of the cell wall (Scheme 8). ETM is one drug that is capable of promoting inhibition in the biosynthesis of this constituent (Fig. 4).

ETM was synthesized in 1960, but was not used in the treatment of TB until 1968. It is important to highlight that the (*S,S*)-ethambutol is the isomeric form used, because the (*R,R*)-ethambutol causes blindness.

The probable action mechanism of ETM involves the inhibition of the arabinofuranosyl transferases, important enzymes that promote the polymerization of arabinose into the arabinan domain of AG[39,40]. The enzyme named *AftA* catalyzes the addition of the first key arabinofuranosyl residue **45** from the sugar donor β-D-arabinofuranosyl-1-monophosphoryldecaprenol (DPA) **44** to the galactan domain of the cell

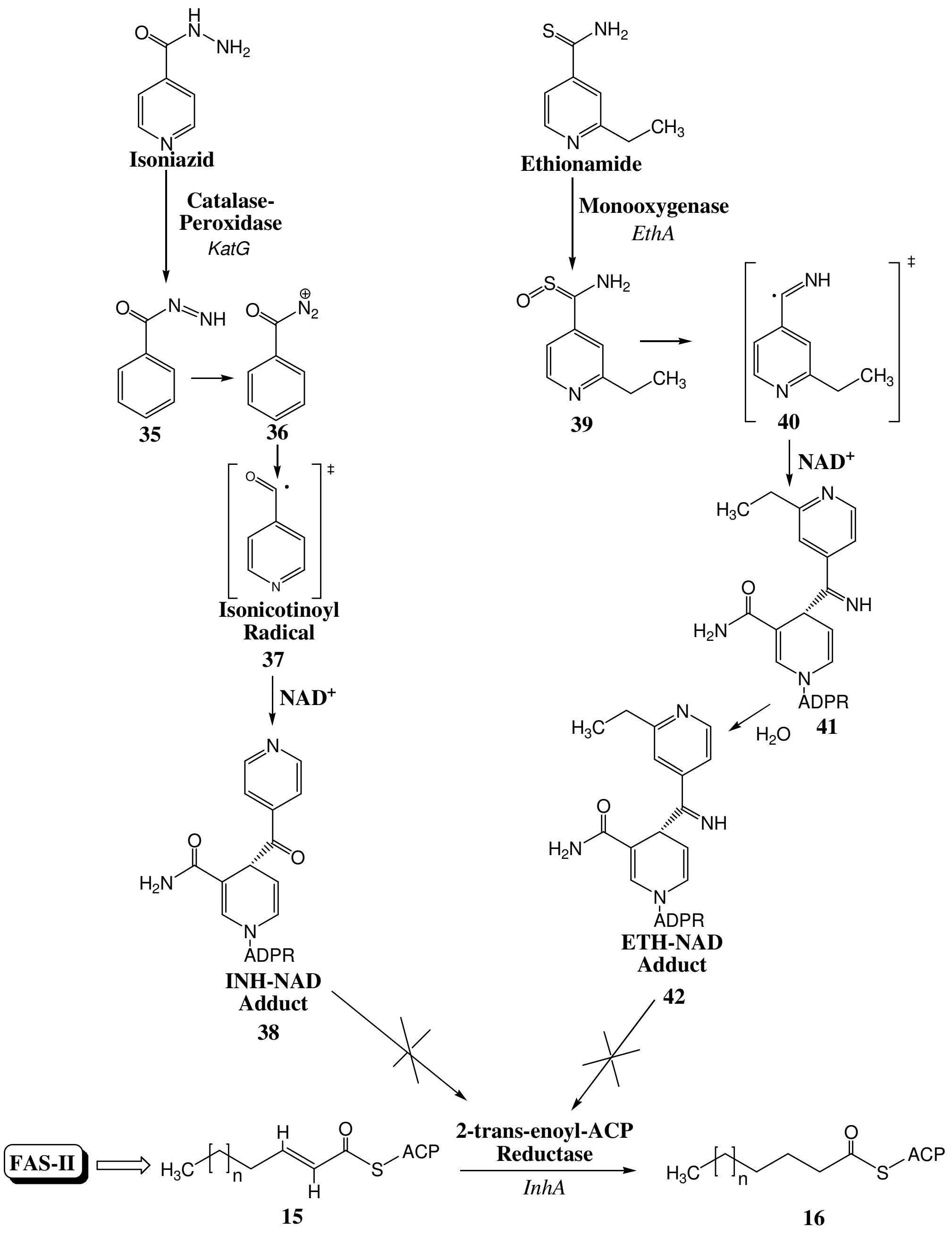

**SCHEME 9.** The probable action mechanism of INH and ETH, which inhibits the biosynthesis of mycolic acids.

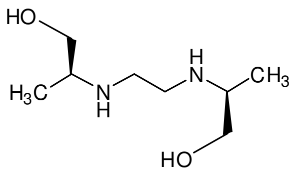

**Ethambutol**

**FIGURE 4.** Structure of ETM.

wall[41,42]. Subsequently, additions are promoted by other arabinofuranosyl transferases, called *EmbA* and *EmbB*, which are inhibited by ETM. With the inhibition of AG synthesis, the formation of the mAGP complex is interrupted and may lead to increased permeability of the cell wall (Scheme 10)[43,44].

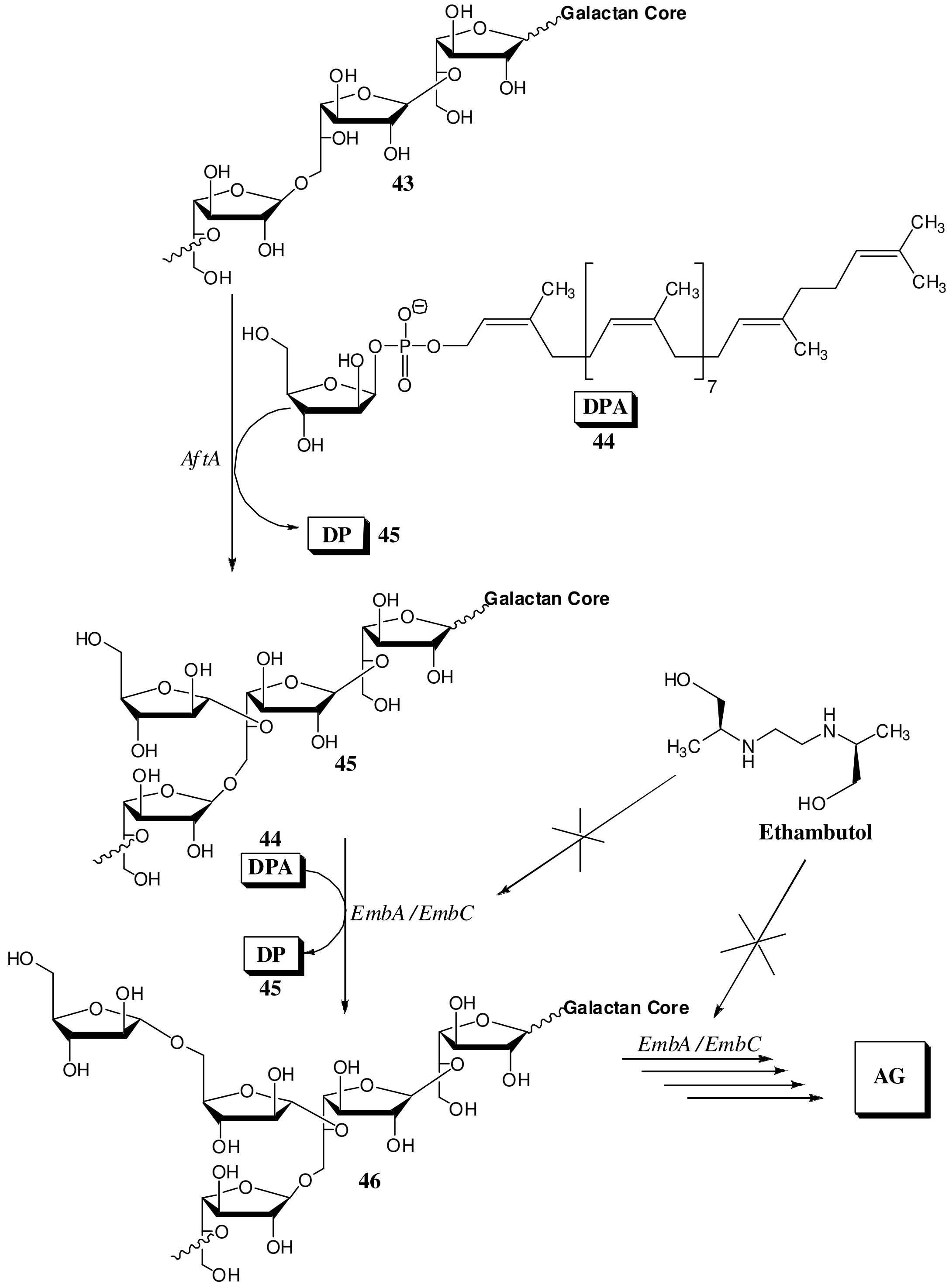

**SCHEME 10.** Proposed action mechanism of ETM, an inhibitor of arabinogalactan biosynthesis.

## SYNTHESIS OF MYCOLIC ACIDS AND ANALOGUES

Mycolic acids and analogues were first prepared by Minnikin and coworkers[45]. For example, mycolic acid analogues **47** (Scheme 11) were synthesized from acetonide **48**, which by Witting reaction with *n*-heptyltriphenyl phosphonium bromide and n-BuLi led to the *cis*-alkene **49**. The next step was based on the modified Simmons-Smith reaction, which allowed the synthesis of the key compound **50**. After the cyclopropanation of the intermediate **49**, the deprotection of the isopropylidene ring in acid condition, followed by oxidative cleavage of the diol by aqueous sodium periodate, furnished aldehyde **51**. The introduction of the ester or acid chain was made in the presence of respective acid or ester phosphoranes and bases, leading to alkenes **52**. After hydrogenation of the double bond and esterification, the mycolic analogues **47** are produced (Scheme 11).

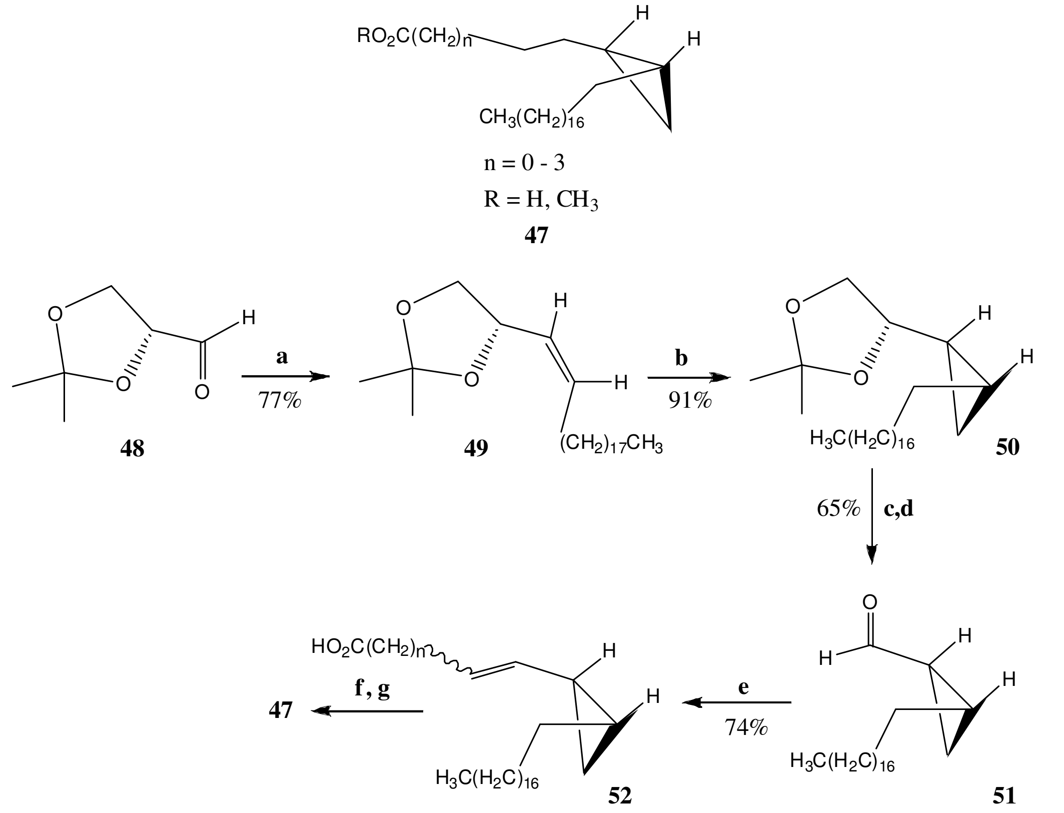

**SCHEME 11.** Synthesis of mycolic acid analogues **47** proposed by Minnikin and coworkers. (a) $Br^-$ $Ph_3P^+(CH_2)_{18}Me$, *n*-BuLi THF,–78°C; (b) $Et_2Zn$ $ClCH_2I$, 1,2-dichloroethane -30°C; (c) HCl, MeOH; (d) $NaIO_4$, $H_2O$; (e) (i) $Ph_3P{=}CHCO_2Me$, MeOH; (ii) $Br^-Ph_3P^+(CH_2)_3CO_2H$; NaHMDS, THF, –78°C; (iii) $Br^-Ph_3P^+(CH_2)_4CO_2H$, NaHMDS, THF, –78°C; (f) $N_2H_4.H_20$, $NaIO_4$, $CuSO_4$, *i*-PrOH. $CH_3CO_2H$, 57°C; (g) DOWEX $H^+$, MeOH, reflux.

Another work in the mycolic acid field made by Minnikin and coworkers was a convenient synthesis of (*Z*)-tetracos-5-enoic acid **56** and racemic *cis*-4-(2-octadecylcyclopropane-1-yl)-butanoic acid **57** (Scheme 12)[46]. These acids are important in the study of biosynthetic pathways of mycolic acids, which suggests that the $\Delta^5$-desaturation of **56** is a key initial step. For example, studies made by Minnikin and coworkers showed that mycolic acid synthesis in extracts of *M. smegmatis* was stimulated by the acid **56,** however strongly inhibited by the cyclopropanation to the ester **57**. The synthesis of **57** was efficiently accomplished based on 1-eicosene **53** as starting material, which after epoxidation by *m*-chloroperoxybenzoic acid and cleavage in presence of *ortho*-periodic acid furnished the aldehyde **54** in

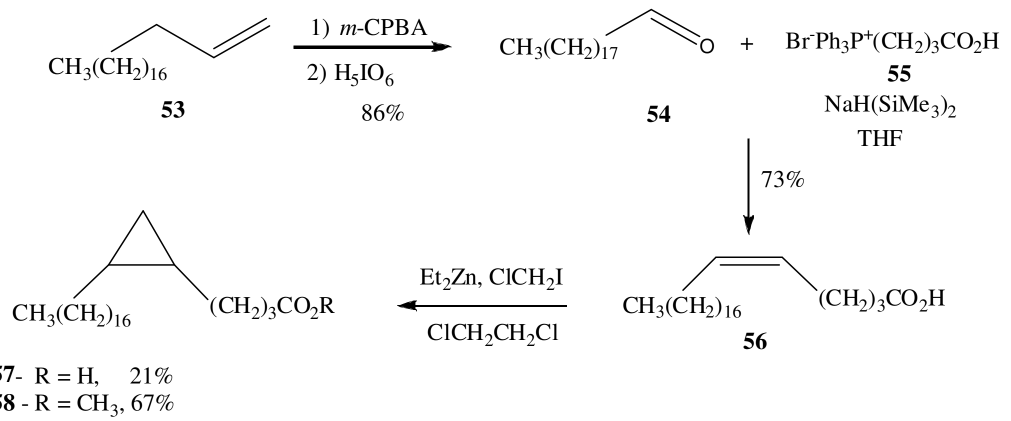

**SCHEME 12.** Synthesis of (*Z*)-tetracos-5-enoic acid **56** and racemic *cis*-4-(2-octadecylcyclopropane-1-yl)-butanoic acid **57** proposed by Minnikin and coworkers.

86% yield (two steps). This aldehyde was coupled by Wittig reaction with the phosphonium salt 4-carboxybutyltriphenylphosphonium **55** leading to the desired (*Z*)-tetracos-5-enoic acid **56** in 73% yield. The cyclopropyl analogues were prepared as a mixture of acid **57** and ester **58** by using Simmons-Smith reaction.

Minnikin and coworkers also synthesized a (1′*R*, 2′*S*) ω-19 cyclopropane fatty acid containing 23–26 carbon chain–length range, which is related to mycobacterial mycolic acids (Scheme 13)[47]. For example, the 24-carbon ω-19 cyclopropane ester **64** was prepared by chain elongation of the 23-carbon ester, which was prepared from D-manitol by the same methodology used by Minnikin mentioned above. The chain elongation was based on classic reactions, beginning with the reduction of ester group, tosylation of alcohol **61**, introduction of cyano group followed by hydrolysis, and finally, esterification of acid **63** to furnish ester **64**.

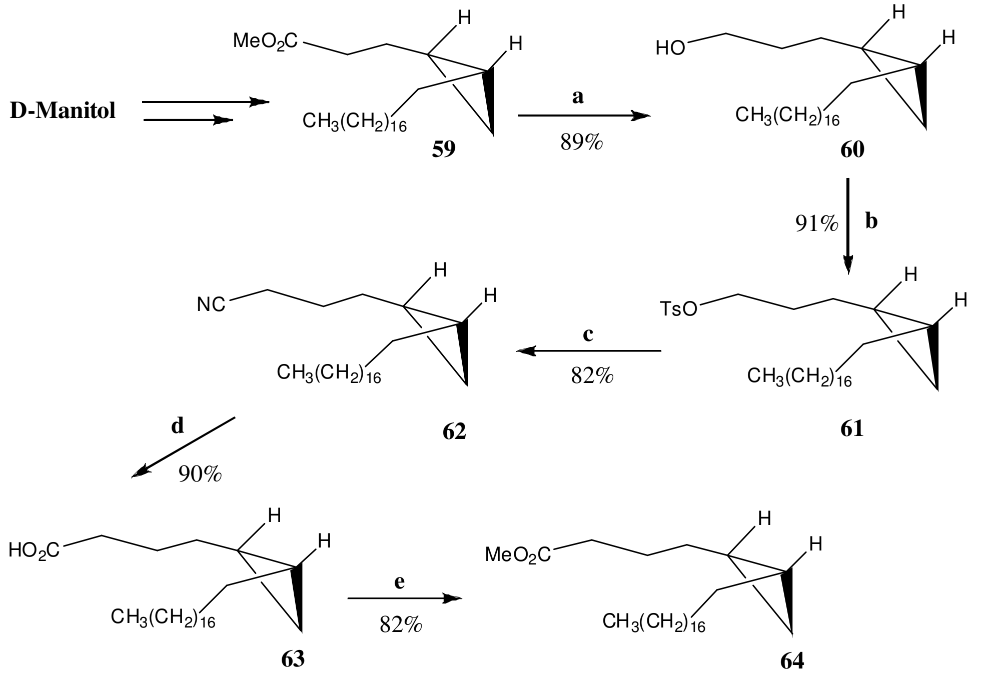

**SCHEME 13.** Synthesis of (1′*R*, 2′*S*) ω-19 cyclopropane fatty acid containing 23–26 carbon chain–length range proposed by Minnikin and coworkers. (a) $LiAlH_4$, THF, reflux, 1 h; (b) TsCl, pyridine, 0°C-r.t.; (c) NaCN, DMSO, 120°C, 2 h; (d) NaOH, EtOH, reflux, 3 h; (e) DOWEX - $H^+$, MeOH, reflux, 3 h.

An important contribution in the mycolic acid field was also made by Baird and coworkers[48], who prepared an elegant synthesis of a mycolic acid **65** (Fig. 5). This deprotected compound was isolated from *M. tb.*

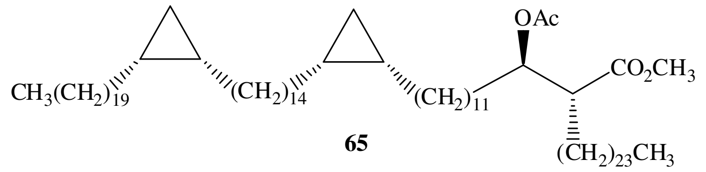

**FIGURE 5.** Mycolic acid 65 synthesized by Baird and coworkers.

The synthetic strategy of Baird for the preparation of mycolic acid **65** (Schemes 14–19) was based on construction method of the cyclopropyl ring used by Minnikin. In this context, the key intermediate (1*R*, 2*S*)-aldehyde **75** was prepared as shown in Scheme 15. First, the intermediate **71** was coupled with the ylid **76** obtained by treatment of **68** with n-BuLi. Compound **68** was prepared by two different methods as shown in Scheme 14. Compound **72** was transformed in aldehyde **74** (three steps), which was treated with 1-carbomethoxy-12-triphenylphosphoniumundecyliodide **77** in the presence of sodium methoxide, followed by reduction, hydrogenation, and oxidation to furnish **75**.

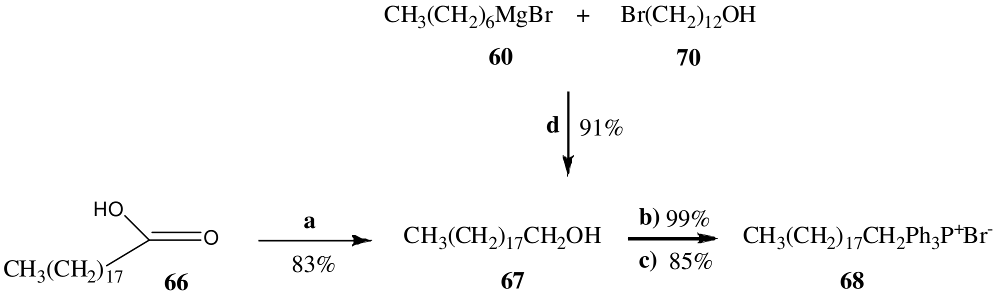

**SCHEME 14.** Synthesis of fragment **68** for the preparation of mycolic acid **65**. (a) $LiALH_4$, THF; (b) aq. HBr $Bu_4N^+Br^-$; (c) $PPh_3$, toluene; (d) $Li_2CuCl_4$, THF.

For the preparation of compound **81** bearing two cyclopropane rings (Scheme 16), the same synthetic strategy was used for the construction of the previous cyclopropyl aldehyde **75** (Scheme 15). However, the Wittig reaction coupling was changed by Julia reaction. First, the ester **78** was converted to sulfone **79** by reaction with 2-mercaptobenzothiazole, triphenylphosphine, and diethyl azodicarboxylate, followed by oxidation of the thioether intermediate. Sulfone **79** was coupled with **75** using a modified Julia reaction, which led to a 1:1 mixture of *E*- and *Z*-alkenes **80**, which, after reduction of carboxylic group and hydrogenation of double bond, furnished the alcohol **81**.

The hydroxyl acid portion represented by other key intermediate **88** was prepared based on the epoxide **82**, which was obtained from (*R*)-aspartic acid. Compound **82** was opened by Grignard reagent **83**, leading to the di-protected triol **84** (Scheme 17). The later compound, after silylation, deprotection of benzyl group, alcohol oxidation, and esterification, led to the intermediate **85**. Further, the primary hydroxyl group of this intermediate was selectively protected by using a hindered silyl group (TBDPS), followed by treatment with LDA and 1-iodotetracosane **86**, previously prepared as described in Scheme 18, furnishing the hydroxyl ester **87**. Finally, compound **87** was acetylated, the silyl group was removed, and the primary hydroxyl group generated was oxidated, leading to intermediate **88**.

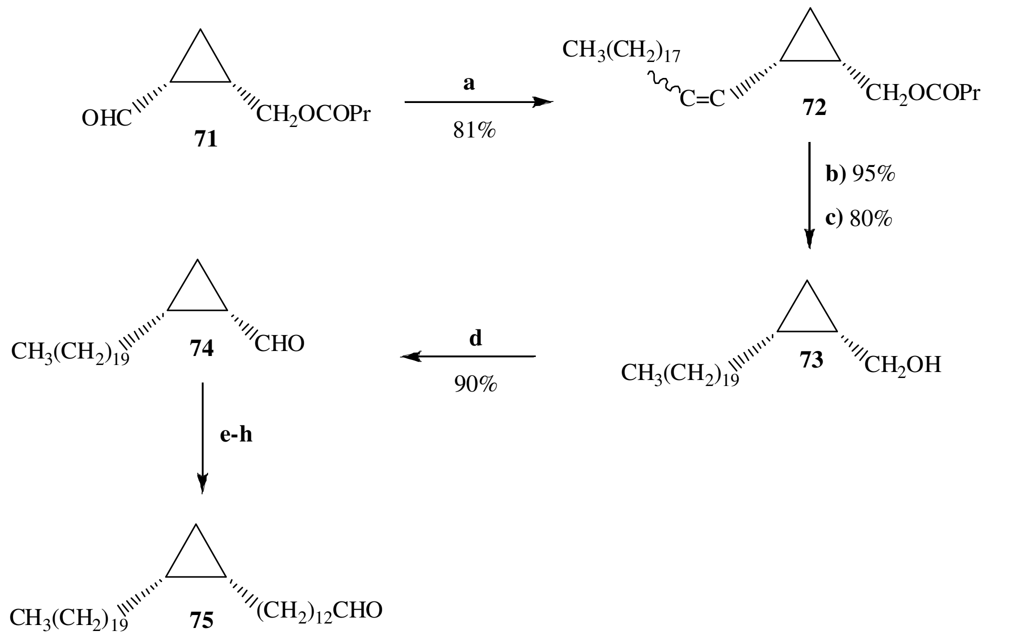

**SCHEME 15.** Synthesis of fragment **75** for the preparation of mycolic acid **65**. (a) $Me(CH_2)_{18}PPh_3Br$, BuLi, THF (81%, 6:1 *Z/E*); (b) $LiAlH_4$, THF; (c) $N_2H_4$, $NaIO_4$, AcOH, $CuSO_4$, *i*-PrOH; (d) PCC, $CH_2Cl_2$; (e) $CH_3O_2C(CH_2)_{11}PPh_3I$ (77), MeONa, THF, DMF (70%); (f) $LiAlH_4$, THF (92%); (g) $N_2H_4$, $NaIO_4$, AcOH, CuSO4, *i*-PrOH (85%); (h) PCC, $CH_2Cl_2$ (93%).

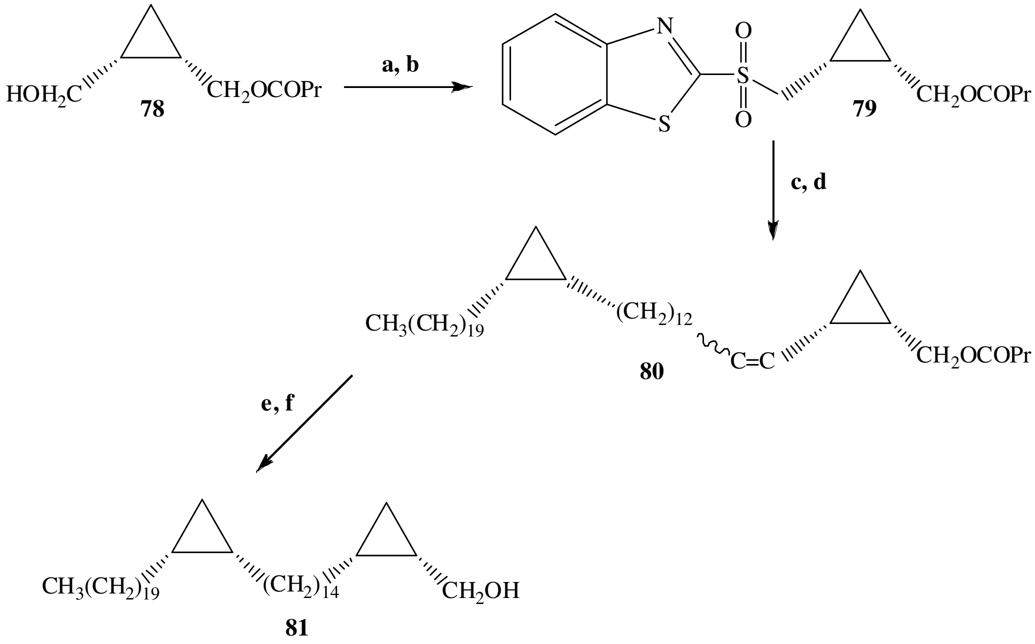

**SCHEME 16.** Synthesis of fragment **81** for the preparation of mycolic acid **65**. (a) 2-Mercaptobenzothiazole, $PPh_3$, DEAD, 81%; (b) $H_2O_2$ or MCPBA, $CH_2Cl_2$; (c) LiHMDS; (d) (**75**) (43% two steps; (e) $LiAlH_4$, THF; (f) $NH_2NH_2$, $NaIO_4$, $CuSO_4$, AcOH, *i*-PrOH (77% two steps).

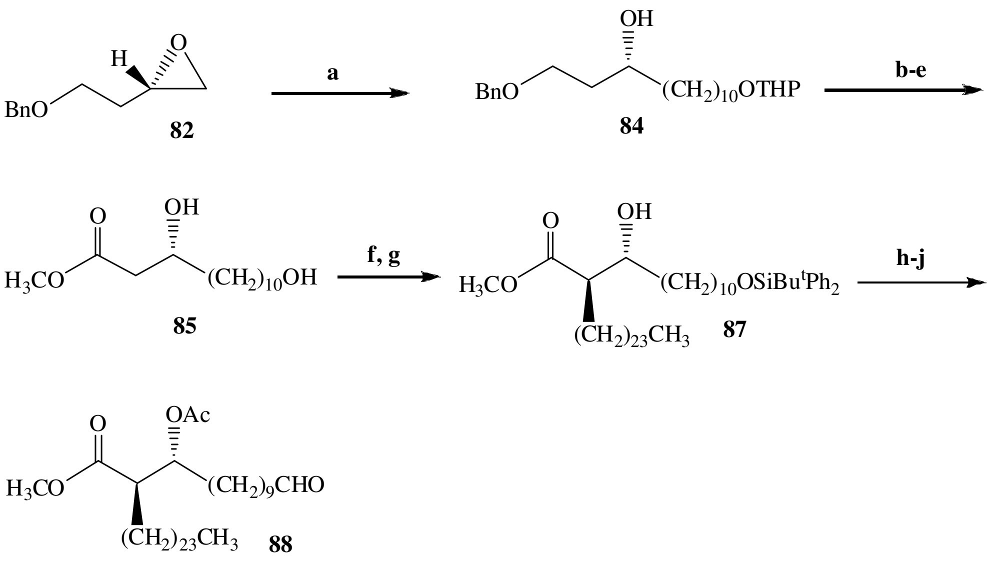

**SCHEME 17.** Synthesis of fragment **88** for the preparation of mycolic acid **65**. (a) $BrMg(CH_2)_9OTHP$ (**83**), CuI, 2 h, –30°C, 86%; (b) imidazole, DMF, $Bu^tSiMe_2Cl$, 93%; (c) $H_2$, Pd/C, MeOH, 84%; (d) $NaIO_4$, $RuCl_3.H_2O$, $CH_3CN$, $H_2O$, $CCl_4$, 69%; (e) MeOH, $H_2SO_4$ (69%); (f) $Bu^tPh_2SiCl$, DMAP, $Et_3N$, 84%; (g) LDA, $CH_3(CH_2)_{23}I$ (**86**), HMPA, 31%; (h) $Ac_2O$, pyridine, 86%; (i) HF.py 75%; (j) PCC, $CH_2Cl_2$ 95%.

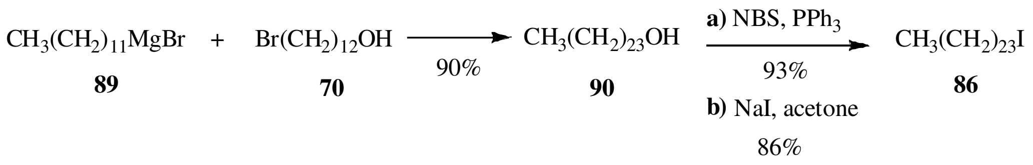

**SCHEME 18.** Synthesis of iodine derivative **86** for the preparation of mycolic acid **65**.

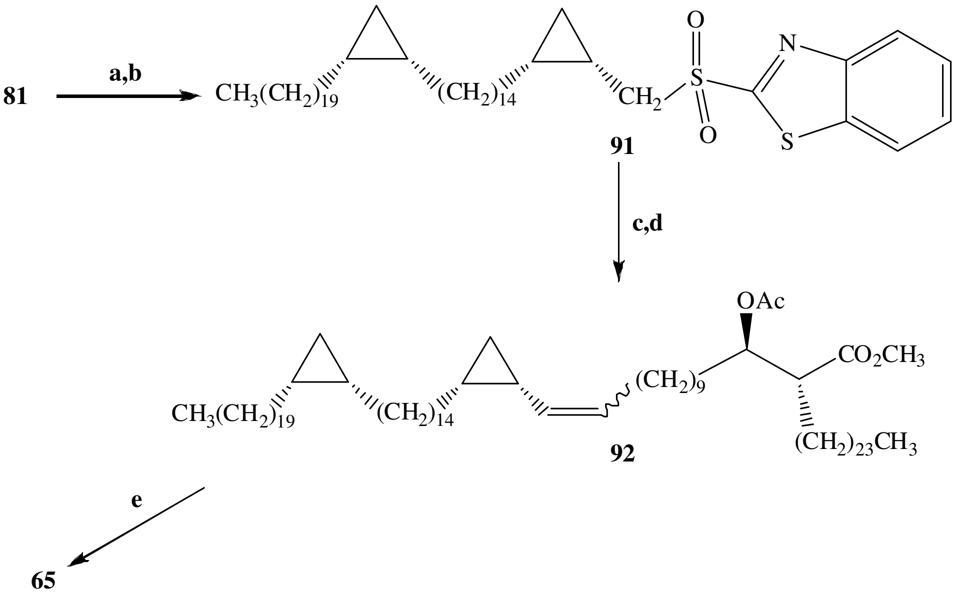

**SCHEME 19.** Final steps for the preparation of mycolic acid **65**. (a) 2-Mercaptobenzothiazole, $PPh_3$, DEAD; (b) MCPBA, $CH_2Cl_2$, 66%; (c) LiBSA; (d) (88); (e) potassium azodicarboxylate, AcOH, THF.

The final steps for the production of the protected mycolic acid **65** is described in Scheme 19. The sulfone **91** was coupled with the aldehyde **88** using Julia reaction conditions, leading to a mixture of *E* and *Z* alkenes **92**, followed by hydrogenation to produce the desired protected mycolic acid **65**.

The synthetic strategy used by Baird and coworkers described above to produce the mycolic acid **65** (Fig. 5) also allowed the synthesis of a different fragment of mycolic acids[49]. For example, Baird and coworkers also synthesized an enantiomer α-methyl-trans-cyclopropane unit **93** (Fig. 6), which is present in different mycolic acids.

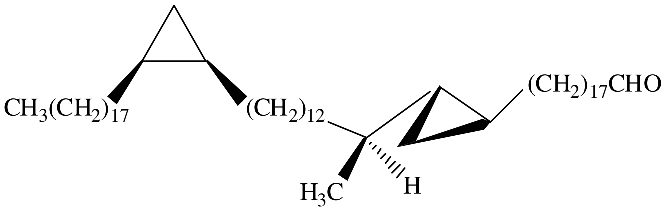

**93**

**FIGURE 6.** Structure of α-methyl-trans-cyclopropane unit **93**.

These authors also developed an improved procedure for the preparation of β-hydroxy-α-alkyl fatty acid **94** (Fig. 7), a fragment of mycolic acids, which could be used for the preparation of different compounds of this class[50]. In this context, the synthesis of fragment **94** (Schemes 20 and 21) started with mono protection of the diol **95**. Oxidation followed by Wittig reaction led to ester α, β-unsaturated **97** (Scheme 20). Sharpless oxidation of intermediate **97** was followed by conversion into cyclic sulfate **99**, which was regioselectively reduced and hydrolyzed to produce the (3*R*)-3-hydroxy ester **100**. The introduction of allyl chain in **100** led to (2*R*,3*R*)-2-allyl-3-hydroxy ester **101**. Secondary alcohol of **101** was protected with silyl group and then conversion to aldehyde **103** was performed by transforming the double bond of **102** into diol group by reaction with $OsO_4$, followed by oxidative cleavage with $NaIO_4$ (Scheme 20). Aldehyde **103** was coupled with sulfone **105** using Julia reaction conditions to furnish compound **106**, which after hydrogenation, cleavage of the pivaloyl group, and oxidation of the alcohol to aldehyde, gave the key intermediate **94** (Scheme 21).

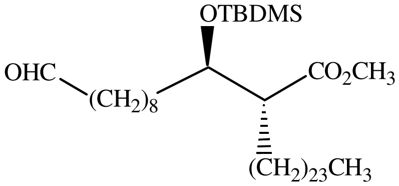

**94**

**FIGURE 7.** Structure of β-hydroxy-α-alkyl fatty acid **94**.

## NEW COMPOUNDS AND CLASSES THAT ACT IN THE *MYCOBACTERIUM TUBERCULOSIS* CELL WALL

Due to the importance of the *M. tb.* cell wall in drug discovery, several research groups have identified between 2005 and 2007 new compounds and classes with promising perspectives.

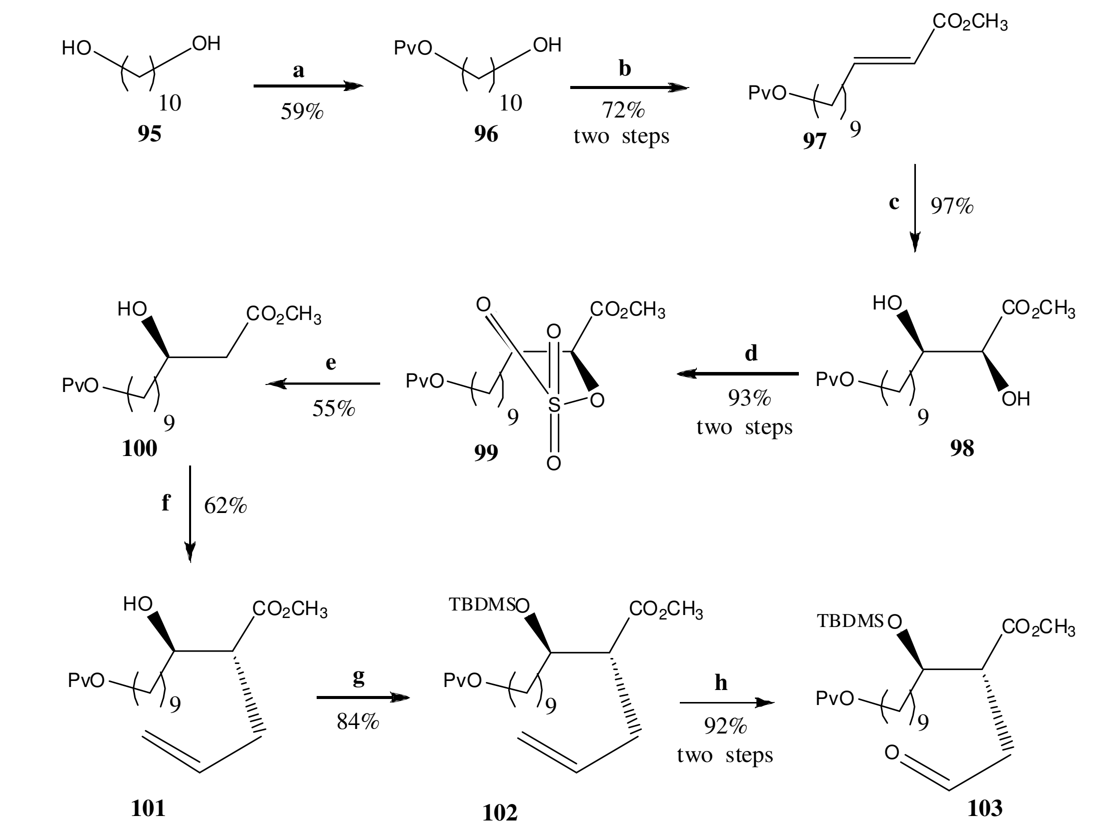

**SCHEME 20.** Synthesis of the intermediate **103** for the preparation of β-hydroxy-α-alkyl fatty acid **94**. (a) (i) PvCl (1.1 mol.equiv.), $Et_3N$ (3 mol.equiv.), THF, $CH_2Cl_2$; (ii) KOH (2 mol.equiv.), MeOH, $H_2O$, (59%); (b) (i) PCC (2 mol.equiv.), $CH_2Cl_2$; (ii) $Ph_3P{=}CHCO_2Me$ (1.1 mol.equiv.), toluene (72%); (c) $(DHQD)_2PHAL$ (1 mol.%), $K_3Fe(CN)_6$ (3 mol.equiv.), $K_2CO_3$ (3 mol.equiv.), $OsO_4$ (4 mol.%), $MeSO_2NH_2$ (1 mol.equiv.), $^t$BuOH, $H_2O$ (97%); (d) (i) $SOCl_2$, (2 mol.equiv.), $CCl_4$; (ii) $NaIO_4$ (1.5 mol.equiv.), $RuCl_3.H_2O$, (5 mol.%) in $CCl_4$, $CH_3CN.H_2O$ (93%); (e) $NaBH_4$ (1 mol.equiv.), *N,N*-dimethylacetamide; $H_2SO_4$, ether, (55%); (f) LDA (2.5 mol.equiv.), THF, $CH_2{=}CHCH_2I$, (1.5 mol.equiv.), HMPA (2 mol.equiv.), THF, (62%); (g) TBDMSCl (1.4 mol.equiv.) imidazole (2.5 mol.equiv.), DMF (84%); (h) (i) $OsO_4$ (4 mol.%), 2,6-lutidine (2 mol.equiv.), (ii) $NaIO_4$ (2.5 mol.equiv.) dioxane, $H_2O$ (92%).

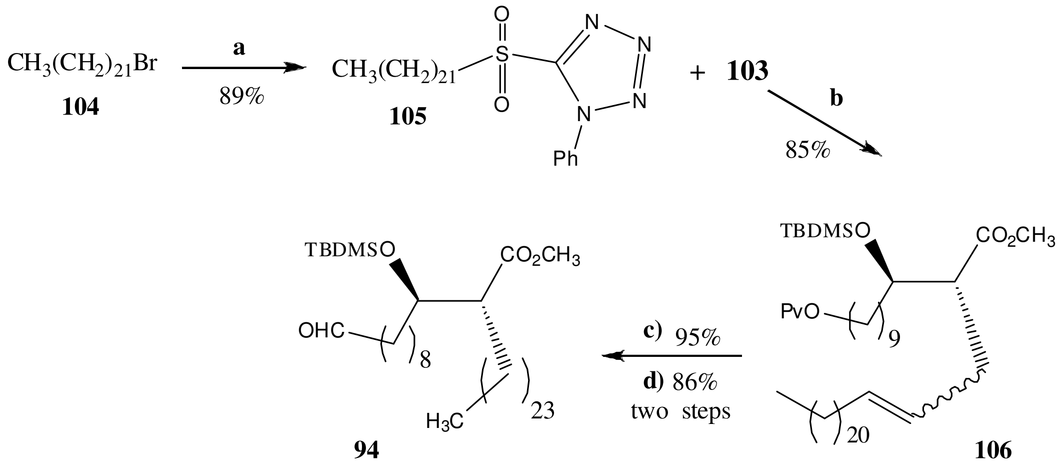

**SCHEME 21.** Final steps for the preparation of β-hydroxy-α-alkyl fatty acid **94**. (a) (i) 1-Phenyl-*1H*-tetrazole-5-thiol (1.05 mol.equiv.), $K_2CO_3$ (1.5 mol.equiv.), acetone; (ii) $H_2O_2$ (47 mol.equiv.), $(NH_4)_6Mo_7O_{24}.4H_2O$ (5.5 mol.%.), THF, IMS (89%); (b) $LiN(Si(Me)_3)_2$ (1.5 mol.equiv.), THF, then 103 (85%); (c) $H_2$, Pt/C, $CH_3CO_2Et$, IMS (95%); (d) (i) KOH, THF, MeOH, $H_2O$; (ii) PCC, $CH_2Cl_2$ (86%).

## Isoniazid Analogues

Despite the role of isoniazid as an essential drug in TB treatment, there are some problems during its utilization, such as side effects, inefficiency against MDR strains, and duration of the treatment. In an attempt to solve these problems, several analogues have been synthesized by different groups[51]. Arora and coworkers[52] recently synthesized a series of isoniazid analogues: compound **107** (Fig. 8) demonstrated similar *in vitro* activity compared to isoniazid against *M. tb.* $H_{37}Rv$ and clinically sensitive strains, as well as superior activity against MDR strains (see Table 2).

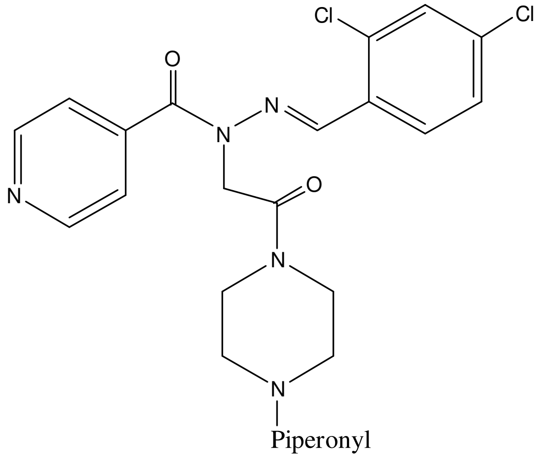

**107**

**FIGURE 8.** Structure of compound **107**.

**TABLE 2**
**Comparative MIC Values (µg/ml) between Compound 107 and Isoniazid**

| Compound | *M. tb.* $H_{37}Rv$ | *M. tb.* Clinically Sensitive | *M. tb.* MDR |
|---|---|---|---|
| 107 | 1.0 | 0.25–0.5 | 2–4 |
| Isoniazid | 0.25 | 0.12–0.25 | 8 – >16 |

## Thiocarlide (Isoxyl) Analogues

The thiourea known as thiocarlide (isoxyl) (Fig. 9) was used in the 1960s to treat TB. However, nowadays, this drug has regained special attention due to its promising *in vitro* antimycobacterial activity between 1–10 µg/ml against different clinical MDR strains[53]. Hence, Besra and coworkers synthesized and evaluated different symmetrical and unsymmetrical analogues of isoxyl against *M. tb.* $H_{37}Rv$ and *M. bovis* BCG with promising results (Fig. 9)[54]. Compounds 1-(*p*-*n*-butylphenyl)-3-(4-propoxyphenyl) thiourea **108** and 1-(*p*-*n*-butylphenyl)-3-(4-*n*-butoxyphenyl) thiourea **109**, in particular, when compared to isoxyl, have demonstrated a nearly tenfold increase in *in vitro* activity in the inhibition of mycolic acid biosynthesis in *M. bovis* BCG. Another important fact provided by these compounds was that their poor inhibition of oleate production indicated that the modifications made in the aromatic ring have an influence in the biological activity.

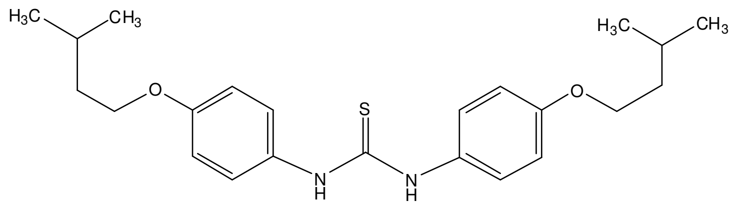

**Isoxyl (Thiocarlide)**

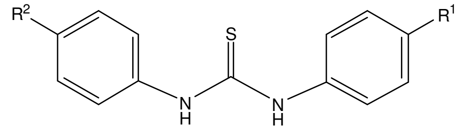

**108** - $R^1$ = $O(CH_2)_2CH_3$ $R^2$ = $(CH_2)_3CH_3$

*M. tb.* $MIC_{99}$ (μg/mL) = <0.1

*M. bovis.* BCG $MIC_{99}$ (μg/mL) = 0.06

**109** - $R^1$ = $O(CH_2)_3CH_3$ $R^2$ = $(CH_2)_3CH_3$

*M. tb.* $MIC_{99}$ (μg/mL) = <0.1

*M. bovis.* BCG $MIC_{99}$ (μg/mL) = 0.7

**FIGURE 9.** Structures of thiocarlide (isoxyl) and compounds **108** and **109**.

## Ethambutol Analogues

An important work published in 2005 in the field of drug discovery was the study of Protopopova and coworkers[55] in collaboration with Clif Barry (NIH/NIAID), which built a library of 63,238 compounds based on 1,2-ethylenediamine, the pharmacophore of ethambutol. These compounds were evaluated against *M. tb.* and 26 demonstrated *in vitro* activity equal to or greater (up to 14-fold) than ethambutol. The compound named SQ109 (Fig. 10) was selected for further development due to its potent activity, MIC 0.7–1.56 μg/ml, an SI of 16.7, and 99% inhibition activity against intracellular bacteria. Additionally, it has demonstrated potency *in vivo* and limited toxicity *in vitro* and *in vivo*.

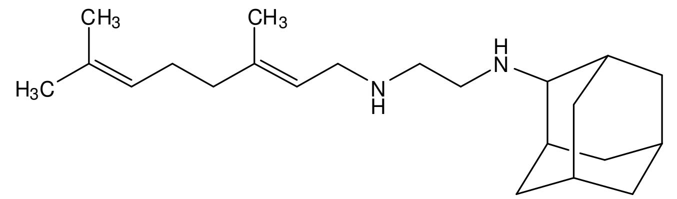

MIC (mg/mL) = 0.7-1.5

SI of 16.7 and 99% inhibition activity against intracellular bacteria

**FIGURE 10.** Structure of compound SQ109.

Pélinski and coworkers synthesized (Scheme 22) and evaluated (Table 3) the anti-TB activity of ferrocenyl ethambutol analogues and ferrocenyl diamines[56]. Preliminary studies demonstrated that the ferrocenyl diamines **111** and **112** displayed good activities against *M. tb.* $H_{37}Rv$. These ferrocenyl analogues were synthesized in 46–77% yield by reacting ferrocene carboxaldehyde **110** with the respective diamines, followed by reduction with $NaBH_4$.

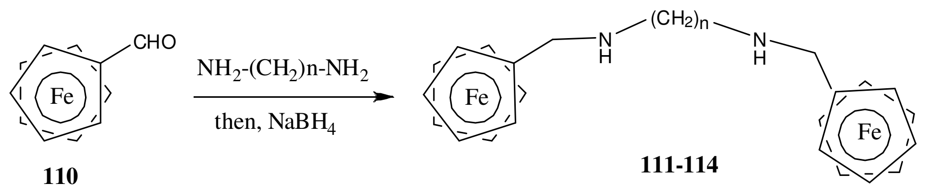

**SCHEME 22.** Structures of ferrocenyl diamines **111** and **112**.

**TABLE 3**
**MIC Values of Compounds 111–114 against *M. tb.***

| N° | n | *M. tb.* MIC (µg/ml) | Overall Yield (%) |
|---|---|---|---|
| 111 | 2 | 8 | 76 |
| 112 | 3 | 8 | 54 |
| 113 | 4 | 32 | 77 |
| 114 | 6 | 32 | 46 |

Tripathi and coworkers described the synthesis and biological evaluation (Scheme 23) of galactopyranosyl amino alcohols as a new class of anti-TB agents[57]. The synthesis was based on the introduction of epichlorohydrin in the diacetonide-α-D-galactose **115**, followed by regioselective oxirane ring opening with several diamines furnishing amino alcohols with two galactopyranosyl units. Compound **117** demonstrated potent *in vitro* activity against $H_{37}Rv$ and MDR *M. tb.* (1.56 µg/ml) when compared with ethambutol (3.25 µg/ml) (Scheme 23).

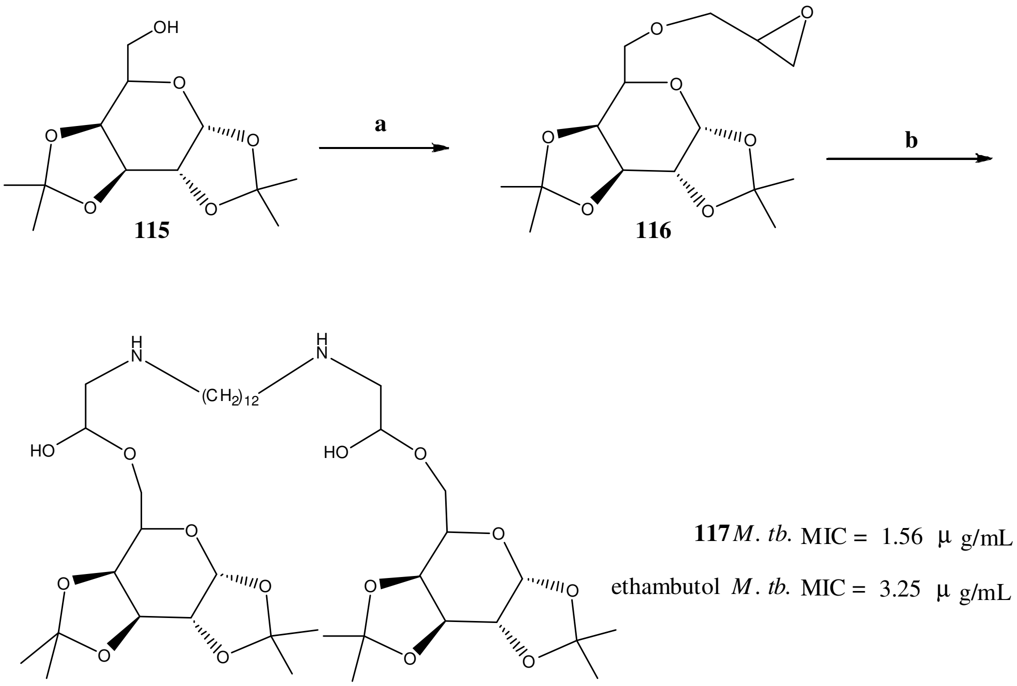

**SCHEME 23.** Synthesis of galactopyranosyl amino alcohol **117**. (a) Epichlorohydrin, aq. NaOH, THF, tetrabutyl ammonium bromide, 40°C, 12 h; (b) $NH_2(CH_2)_nNH_2$; ethanol, 30°C, 12–18 h.

Tripathi and coworkers also described the synthesis (Fig. 11) of glycosylated amino alcohols by classical carbohydrate chemistry, which were evaluated (Table 4) against *M. tb.* $H_{37}Ra$ and $H_{37}Rv$[58]. The most promising compounds were **118** and **119,** presenting MIC ranging from 6.25 to 3.12 µg/ml.

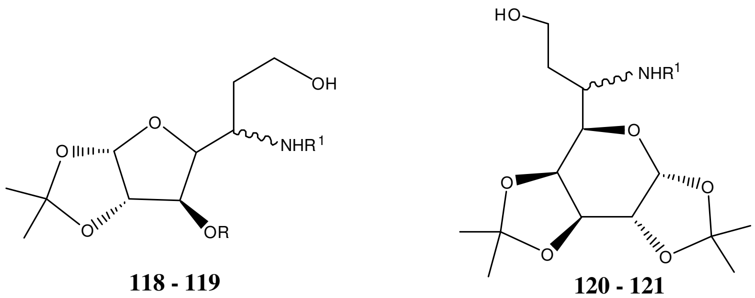

**118 - 119** **120 - 121**

**FIGURE 11.** Structures of glycosylated amino alcohols **118–121**.

**TABLE 4**
**MIC values of Compounds 118–121**

| N° | R | $R^1$ | *M. tb.* MIC (µg/ml) $H_{37}$ Ra | *M. tb.* MIC (µg/ml) $H_{37}$ Rv |
|---|---|---|---|---|
| 118 | $CH_3$ | $(CH_2)_{15}CH_3$ | 6.25 | 6.25 |
| 119 | $CH_2Ph$ | $(CH_2)_{11}CH_3$ | 3.12 | 3.12 |
| 120 | — | Oleyl | 3.12 | 6.25 |
| 121 | — | $(CH_2)_{15}CH_3$ | 25 | 3.12 |

A new class of aryloxyphenyl cyclopropyl methanone derivatives was discovered by Tripathi and coworkers[59], which was synthesized by using one-pot reaction between compound **122** with 12 different alcohols **123** (Scheme 24). Compounds of this class have been evaluated against *M. tb.* $H_{37}Rv$ *in vitro* and presented MICs ranging from 25 to 3.12 µg/ml (Table 5).

## PA-824

Some classes of compounds containing nitro groups are under investigation as anti-TB drugs; the leader is represented as PA-824 (Fig. 12). This nitroimidazole was developed by PathoGenesis Inc. in 1995 for cancer treatment. However, despite its promising TB activity reported in 2000, when Chiron took over PathoGenesis Inc. in 2000, the project was aborted. However, TB Alliance and Chiron signed a license agreement that gave TB Alliance rights to develop this molecule. PA-824 has shown potent *in vitro* activity against *M. tb.* with MIC of 0.03–0.2 µg/ml[60]. This compound also possesses high activity in mice with no toxicity in rodent models, as well as excellent sterilizing activity compared with isoniazid and rifampin. The mechanism of action of this prodrug, which requires activation, is due to a flavinoid known as F-420 cofactor, which activates PA-824, which subsequently inhibits the synthesis of protein and cell wall lipids. Due to its important results, PA-824 is under clinical phase.

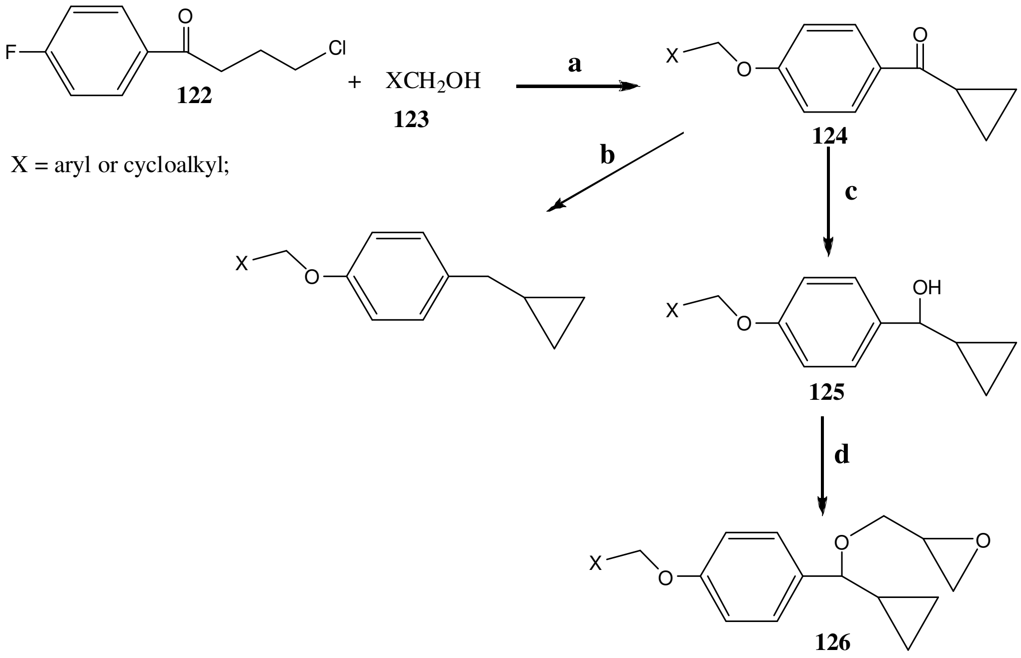

**SCHEME 24.** Structure of aryloxyphenyl cyclopropyl methanone derivative **126**. (a) NaH, THF, TBAB, 0–30°C; (b) $NH_2NH_2.2HCl$, diethylene glycol, 190°C; (c) $NaBH_4$, methanol, 0–25°C; (d) epichlorohydrin, NaH, THF, TBAB, 0–25°C.

**TABLE 5**
***In vitro* Anti-TB Activity of More Active Methanone Derivatives 124–126 (µg/ml) Prepared by Tripathi and coworkers[59]**

| N° | X | MABA *M. tb.* $H_{37}Ra$ | Agar Microdilution *M.tb.* $H_{37}Rv$ |
|---|---|---|---|
| 124 | Phenyl | 25 | 6.25 |
| 125 | 4-Methoxy phenyl | >50 | 3.12 |
| 125 | 4-Fluoro phenyl | 25 | 6.25 |
| 125 | Cyclopentyl | 25 | 3.12 |
| 126 | 4-Fluoro phenyl | nd | 3.12 |

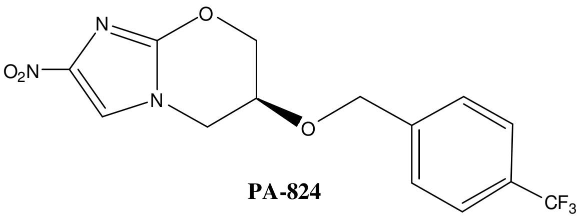

**FIGURE 12.** Structure of PA-824.

## Nitrofuranylamides

Lee and coworkers made a synthesis and biological evaluation (Table 6) of a series of nitrofuranylamides (Fig. 13) as anti-TB agents, with promising results[61]. These results were based on the activity of compound **127**, discovered by a screen for *M. tb.* UDP-Gal mutase (Glf) inhibition, which possesses $IC_{50}$ of 12 μM Glf inhibitor and MIC of 1.6 μg/ml. For example, compounds **127**–**131** (Fig. 13) have been evaluated *in vitro* and *in vivo* with favorable perspectives. It is important to mention that, as mycolic acid, galactofuranose is an essential component of the mycobacterial cell wall, of which UDP-galactofuranose is biosynthesized from UDP-galactopyranose by using the enzyme UDP-galactose mutase[62,63].

**TABLE 6**
***In vitro* anti-TB Activity of Compounds 127–131**

| N° | R | $R^1$ | n | *M. tb.* MIC (μg/ml) | Glf $IC_{50}$ (μ*M*) |
|---|---|---|---|---|---|
| 127 | Cl | $OCH_3$ | 0 | 1.6 | 12 |
| 128 | Cl | H | 0 | 0.8 | 15 |
| 129 | F | H | 0 | 0.8 | 28 |
| 130 | $OCH_3$ | H | 0 | 0.8 | 42 |
| 131 | $OCH_3$ | $OCH_3$ | 1 | 0.2 | 23 |

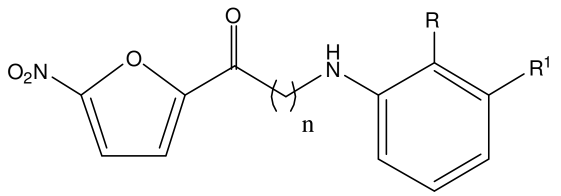

**FIGURE 13.** Structures of nitrofuranylamides **127**–**131**.

## Thiolactomycin

Thiolactomycin 1 (TLM, Fig. 14) is a naturally occurring antibiotic obtained from the fermentation broth of a strain of actinomycetes belonging to the genre *Nocardia* sp., found in a Japanese soil sample and described for the first time in 1982 by Oishi and coworkers[64]. TLM exhibits a broad spectrum of activity *in vivo* against many pathogenic micro-organisms, such as Gram-positive, Gram-negative, and anaerobic bacteria. TLM shows modest activity against laboratory strains of *M. tb.* (MIC = 62.5 μg/ml). However, this natural product possesses relevant activity against several strains of *M. tb.,* which are resistant to other drugs[65,66]. TLM inhibits KasA and KasB, two enzyme components of the specialized FAS II system involved in the synthesis of the very long-chain meromycolic acids, constituent of building blocks for bacterial cell walls[67,68].

The physical and pharmacokinetic properties of TLM, such as low molecular weight (MW 210 g/mol), high water solubility, appropriate lipophilicity (logP = 3), good oral absorption, and low toxicity profile in mice[69] made it an attractive lead molecule for TB treatment[70]. The success of TLM has increased the efforts towards the synthesis of even more effective agents, leading to the current list of compounds that present a good level of activity and can be seen as promising drugs in curing TB disease.

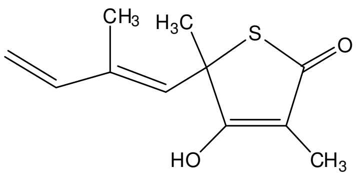

**Thiolactomycin**

**FIGURE 14.** Structure of thiolactomycin (TLM).

The most common synthetic route for the synthesis of thiolactone ring is described by Wang and Salvino (Scheme 25)[71]. This method involves Claisen self-ester condensation of methyl propionate **132** to generate ß-keto ester **133**. Selective bromination of **133**, followed by nucleophilic substitution of bromine by thioacetic acid gave thioester **135**. Finally, cyclization in basic conditions furnished the thiolactone **136**, the key intermediate for the synthetic approach to thiolactomycin analogues.

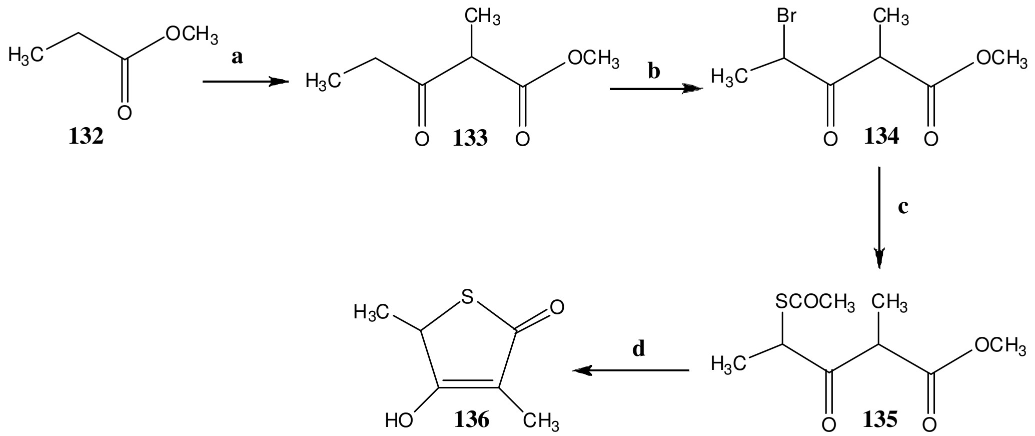

**SCHEME 25.** Synthesis of thiolactone **136**. (a) Anhydrous KH, THF; (b) $PyHBr_3$, acetic acid; (c) AcSH, $Et_3N$, $CH_2Cl_2$; d) KOH, $H_2O$-EtOH.

Recently, Senior and coworkers[72] described the preparation of a series of TLM analogues (Scheme 26). The synthesis was based on the introduction of propargyl group in the C-5 position of the thiolactone **136**, followed by Sonogashira coupling reaction, producing compounds **138a-c** in 47–86% yields. These compounds exhibit higher *in vitro* inhibitory activity against the recombinant *M. tb.* ß-ketoacyl-ACP synthase mtFabH condensing enzyme (Table 7) when compared to TLM.

Kamal and coworkers synthesized (Scheme 27) and evaluated (Table 8) the anti-TB activity of a series of thiolactomycin analogues[73]. Preliminary studies have demonstrated that analogues **140** and **141** displayed moderate activities against *M. tb.* $H_{37}Rv$ and clinical isolates (sensitive strains), however, significant activity against MDR strains. These thiolactomycin analogues have been synthesized from thiolactone **136** by the etherification of hydroxy group with alkyl dihalides to afford compounds **139** and **140**. The bromo ether derivative **140** was treated with methylthioglycolate to produce compound **141**.

Starting from the crystal structure of *M. tb.* antigen 85C, Gobec and coworkers[74] designed and synthesized a series of phosphonate inhibitors of its mycolyltransferase activity. The inhibitors, comprised of a phosphonate moiety, mycolic acid mimetic, and a trehalose surrogate, were tested for their inhibition of recombinant *M. tb.* antigen 85C mycolyltransferase activity and for *in vitro M. avium* growth inhibition assay. Ethyl 3-phenoxybenzyl butylphosphonate **142** and (1,3-dioxo-1,3-dihydro-2H-isoindol-2-yl) methyl ethyl heptylphosphonate **143** inhibited the mycolyltransferase activity of antigen 85C with

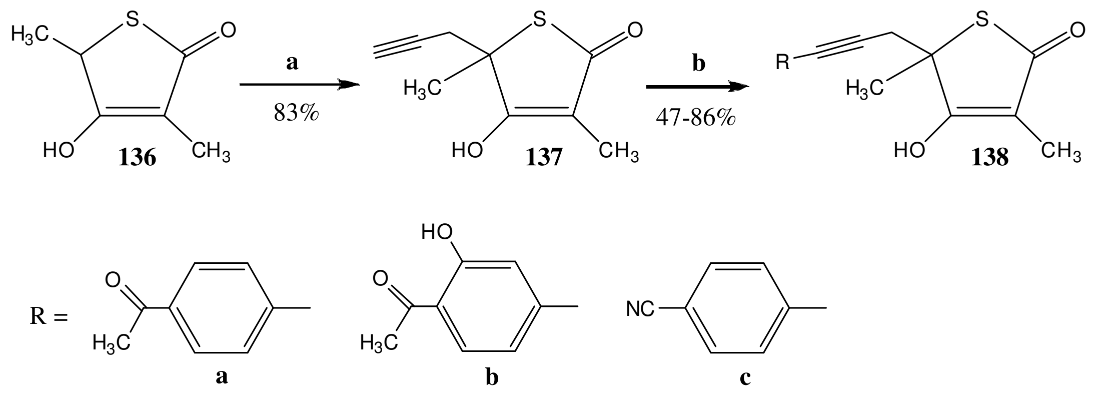

**SCHEME 26.** Synthesis of thiolactone **138**. (a) LHMDS, $HCCCH_2Br$, –78°C; (b) DMF, $Et_3N$, CuI, $(PPh_3)_2PdCl$, R-I.

**TABLE 7**
***In vitro* mtFabH Activity**

| Compounds | $IC_{50}$, μ*M* (mtFabH) |
|---|---|
| TLM | 74.9 |
| 138a | 4 ± 0.4 |
| 138b | 7 ± 0.2 |
| 138c | 7 ± 0.5 |

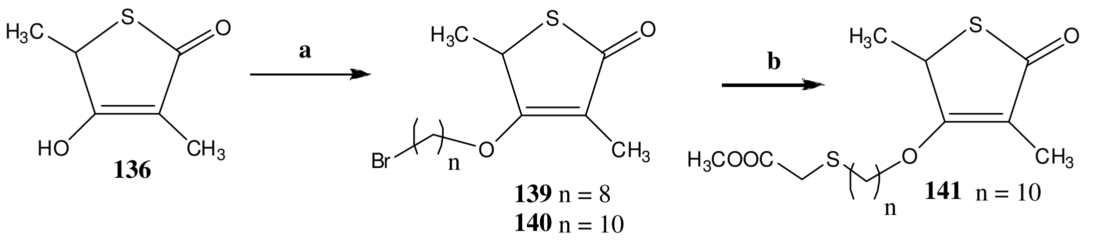

**SCHEME 27.** Synthesis of thiolactone **141**. (a) $Br(CH_2)_nBr$, anhydrous $K_2CO_3$, acetone, reflux; (b) $CH_3OOCCH_2SH$, anhydrous $K_2CO_3$, acetone, reflux.

**TABLE 8**
**MIC Values (μg/ml) of Compounds 140 and 141 and Isoniazid**

| Compound | *M. tb.* $H_{37}Rv$ | *M. tb.* Clinically Sensitive | *M. tb.* MDR |
|---|---|---|---|
| 140 | 4.0 | 4.0–16.0 | 4.0–16.0 |
| 141 | 1.0 | 1.0–4.0 | 1.0–4.0 |
| Isoniazid | 0.25 | 0.12–0.25 | 8 – >16 |

$IC_{50}$ in the low micromolar range. Compound **142** proved to be the most promising compound, inhibiting the antigen 85C with an $IC_{50}$ value of 2.0 µ*M* and showing substantial *in vitro* inhibition of growth of *M. avium*. Whereas compound **143** was the most potent inhibitor of mycolyltransferase activity ($IC_{50}$ = 1.3 µ*M*), it showed no detectable activity against *M. avium* in the *in vitro* assay (Fig. 15) .

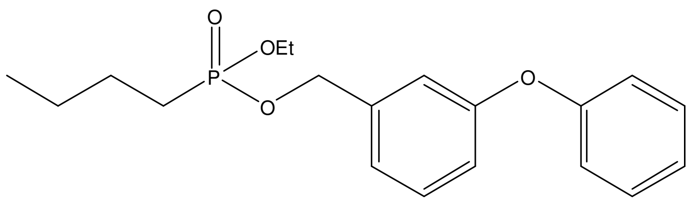

**142**
$IC_{50}$=2.0µM; MIC= 248.8±129.8µg/ml

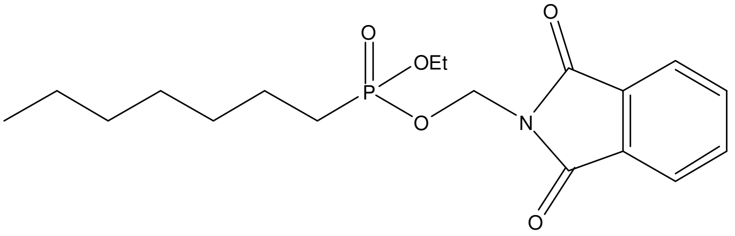

**143**
$IC_{50}$=1.3µM; MIC= inactive

**FIGURE 15.** Structures of compounds **142** and **143**.

## CONCLUSION

Because TB has become a worldwide problem again, there is an urgent need for new drugs and strategies to fight against this important infectious disease. In this context, the mycobacterial cell wall regains attention, and due to modern analysis techniques and the elucidation of the *M. tb.* Genome, there is now a better comprehension of the different aspects of the cell wall of *M. tb.*, such as structure, biosynthesis, and genetics aspects. Considering the latter and the importance of mycolic acids for maintenance of the integrity of the mycobacterial cell wall, this class of compounds has become an attractive cellular target for the development of novel drugs against TB.

## ABBREVIATIONS

**Ac** = Acetyl; **$AccD_4$/$AccD_5$** = Acyl-CoA carboxylases 4 and 5; **ACP** = Acyl carrier protein; **AG** = Arabinogalactan; **AG-M** = Arabinogalactan-mycolate; **Ag85** = Antigen 85; AIDS = Acquired immune deficiency syndrome; **AMP** = Adenosine monophosphate; **BCG** = Bacillus Calmette-Guérin; ***n*-Bu** = *n*-buthyl; **DE** = β-hydroxyacyl dehydrase; **DEAD** = Diethyl azodicarboxylate; **$(DHQD)_2$PHAL** = Hydroquinidine 1,4-phthalazinediyl diether; **DMAP** = 4-Dimethylaminopyridine; **DMF** = *N,N*-Dimethylformamide; **DMSO** = Dimethyl sulfoxide; **DA** = Arabinofuranosyl residue; **DPA** = β-D-arabinofuranosyl-1-monophosphoryldecaprenol; **ER** = Enoyl reductase; **ETH** = Ethionamide; **ETM** = Ethambutol; **Et** = Ethyl; **FAS-I** = Fatty acid synthase type I; **FAS-II** = Fatty acid synthase type II; **HMDS** = Hexamethyldisilazane; **HMPA** = Hexamethylphosphoramide; **$IC_{50}$** = Half maximal inhibitory; **IMS** =

Methylated spirits ("metho") is a mixture of ethyl alcohol (95%) and methyl alcohol (%5); **INH** = Isoniazid; **KAS** = β-ketoacyl synthase; **KR** = β-ketoacyl reductase; **LDA** = Lithium di-isopropylamide; **LiBSA** = Lithium *N*, *O*-Bis(trimethylsilyl)acetamide; **mAGP** = Mycolyl-arabinogalactan–peptidoglycan; **MCPBA** = *m*-Chloroperoxybenzoic acid; **MDR** = Multidrug resistant; **Me** = Methyl; **MIC** = Minimum inhibitory concentration; **MS** = Mass spectrometry; **MST** = Mean survival time; ***M. tb.*** = *Mycobacterium tuberculosis*; **Myc-PL** = 6-*O*-mycolyl-β-D-mannopyranosyl-1-phosphoheptaprenol; **NAD$^+$** = Nicotinamide adenine dinucleotide; **NADH** = Reduced form of NAD+; **NADPH** = Nicotinamide adenine dinucleotide phosphate; **NBS** = *N*-Bromosuccinimide; **NMR** = Nuclear magnetic resonance; **Pks 13** = Polyketide synthase 13; **PCC** = Pyridinium chlorochromate; **Ph** = Phenyl; **Pv =** Pivaloyl; **Py** = Pyridine; **SAM** = S-Adenosyl-L-methionine; **TB** = Tuberculosis; **TBAB** = Tetra *n*-butyl ammonium bromide; **TBAF** = Tetra *n*-butyl ammonium fluoride; **TBDMSCl** = *t*-Butyldimethylsilyl chloride; **TDM** = Trehalose dimycolate; **TML** = Thiolactomycin; **TMM** = Trehalose monomycolate; **THF** = Tetrahydrofuran; **Ts** = Tosyl.
